# Cancer Prevention Clinical Trials: Advances and Challenges

**DOI:** 10.3390/cancers18030390

**Published:** 2026-01-27

**Authors:** Elizabeth R. Francis, Farzeen Z. Syed, Arun Rajan, Eva Szabo

**Affiliations:** 1 Thoracic and GI Malignancies Branch, Center for Cancer Research, Cancer Prevention Fellowship Program, Division of Cancer Prevention, Occupational and Environmental Epidemiology Branch, Division of Cancer Epidemiology and Genetics, National Cancer Institute, National Institutes of Health, Bethesda, MD 20892, USA; elizabeth.francis@nih.gov; 2 Thoracic and GI Malignancies Branch, Center for Cancer Research, National Cancer Institute, National Institutes of Health, Bethesda, MD 20892, USA; farzeen.syed@nih.gov; 3 Thoracic and GI Malignancies Branch, Center for Cancer Research, Center for Immuno-Oncology, National Cancer Institute, National Institutes of Health, Bethesda, MD 20892, USA; rajana@mail.nih.gov; 4 Thoracic and GI Malignancies Branch, Center for Cancer Research, Lung and Upper Aerodigestive Cancer Research Group, Division of Cancer Prevention, National Cancer Institute, National Institutes of Health, Bethesda, MD 20892, USA

**Keywords:** cancer prevention, precision prevention, clinical trials, immunoprevention, premalignancy, germline pathogenic variants as targets

## Abstract

Prevention of cancer is an appealing strategy to reduce the burden of illness associated with cancer, but despite the rapidly advancing understanding of the early phases of carcinogenesis, translation of biologic insights into actionable public health strategies has been challenging. Phase III clinical trials have historically required large numbers of participants and lengthy durations to show effects in the minority of participants who develop cancer during the finite span of each trial. Early-phase trials help to refine intervention strategies and provide preliminary human safety and efficacy data to justify phase III trials. Recent advances in trial methodology and developments in immunopreventive strategies have energized the field of cancer prevention and provide potential paths for prevention of multiple cancer types. In this review we discuss the history and current state of cancer prevention trials, with a focus on overcoming inherent biologic and methodologic barriers to preventive agent development.

## Simple Summary

Cancer affects millions worldwide, and preventing the disease before it develops is a more favorable approach than treating established cancer. Despite significant advances in understanding how cancer forms, creating effective prevention strategies has proven challenging. Prevention trials require large populations followed for many years to show benefit, which is both costly and complex. Recent innovations in trial design and immune-based prevention methods offer new opportunities to overcome these obstacles. This review examines the challenges in cancer prevention and the emerging strategies that could transform prevention from theory into practice.

## 1. Introduction

While recent decades have witnessed immense progress in the treatment and early detection of cancer, reducing the global burden of cancer remains a daunting challenge [1]. Demographic predictions indicate that by 2050, there will be 35 million new cancer cases worldwide, so substantial investments in prevention, to include risk factor mitigation (e.g., smoking, alcohol, overweight/obesity, and infection), are needed to avert this large number of cancer cases and deaths. A multi-pronged approach to targeting the process of carcinogenesis throughout its life cycle, from initiation through metastasis, is understood to be key to making cancer a manageable chronic disease.

However, after the initial enthusiasm of targeting the carcinogenic process when it is less complex and accompanied by less immunosuppression than metastatic disease, the inherent difficulties in preventing ‘that which is not yet present’ have become evident. Cancer treatment has evolved to target the vastly different biological processes giving rise to different cancer types (e.g., targeting a variety of actionable driver mutations in non-small cell lung cancer and other cancers) to harness the power of genomic analysis to bring precision oncology to clinical practice. In an analogous fashion, precision prevention aims to use the mechanistic understanding of the carcinogenic process to bring preventive strategies to individuals who are most likely to benefit from the interventions and less likely to experience negative effects [2], albeit without the benefit of easy genomic analysis to assess which pathway to carcinogenesis is or will be occurring in a given individual.

The field of cancer prevention is at a crossroads. The promise of a rapidly increasing understanding of carcinogenesis has identified a number of gaps that need to be addressed prior to successful translation to routine clinical care. As discussed below, carcinogenesis is a complex process occurring via different mechanisms and at different rates throughout the body. The incomplete understanding of this process and the inability to assess the extent of ongoing carcinogenesis in any given individual at any given time limit our ability to identify rational prevention targets and to precisely match individuals with the right interventions. Risk assessment often requires invasive procedures to identify individuals at high risk for cancer and to assess the effect of interventions on biomarkers of malignancy. These procedures are generally not part of the standard of care, complicating accrual to clinical trials and adding significantly to trial cost. There is a need to develop noninvasive biomarkers, both for risk and endpoint assessment. Furthermore, there is a lack of validated surrogate endpoints to replace cancer incidence and thereby shorten trial duration. In this review, we summarize the challenges facing the development of interventions to target ongoing carcinogenesis, and we discuss recent advances with the potential to bring interventions to public health benefit.

## 2. Challenges in Cancer Prevention Trial Design

### 2.1. Biological Complexity

The challenges of cancer prevention begin with the fundamental biology of carcinogenesis. Carcinogenesis is inherently a heterogeneous process driven by multiple independent clones that coexist within the same anatomical region, creating a field of genetically and epigenetically altered epithelium [3]. Not only is there heterogeneity in the driving processes that lead to cancers arising in different organs and between individuals with a cancer arising from the same organ, but there is also considerable evolutionary clonal variation within each individual [4]. In head and neck mucosa, for example, carcinogen exposure produces a broad field of injury in which distinct premalignant clones develop and evolve independently, making it difficult to target a single lesion or pathway [5]. This concept of field cancerization extends across multiple organ systems and reflects widespread molecular alterations that are not clinically visible or necessarily targetable by the same intervention [6]. As a result, sampling a single lesion often fails to capture the biological diversity present within the entire field. Furthermore, spatial patterns of early cancer growth demonstrate that premalignant lesions may contain multiple subclones with different evolutionary trajectories and signaling profiles, forming a mosaic of risk rather than a uniform premalignant population [7]. Even within a single field, genetically distinct clones diverge early, potentially making the response to a single targeted preventive agent highly variable [5].

Biological heterogeneity also makes it far more difficult to evaluate efficacy in prevention than in treatment. Cancer incidence endpoints occur years after the intervention begins, and imaging does not reliably capture early biological changes. As a result, prevention trials rely heavily on mechanistic understanding, animal models, and epidemiologic signals to identify rational targets [8]. Where possible, efforts focus on biologically defined high-risk groups where initiating pathways are clear, such as individuals with germline syndromes or oncogenic drivers, because the known initiating mutation provides a more precise and potentially actionable target for prevention.

In summary, this heterogeneity has profound implications for preventive trial design and successful intervention, particularly in non-germline cohorts. As difficult as it is to obtain at-risk or premalignant tissue (which often requires invasive procedures to obtain tissue), there is a real possibility that tissue sampling may miss the pertinent molecular target(s). Interventions will need to be broad enough to account for clonal evolution, which suggests that targeting single molecular abnormalities or pathways downstream of germline mutations or oncogenic drivers may be difficult to accomplish without the emergence of resistance bypass mechanisms. Furthermore, since phase II trial designs depend on assessing the effect of an intervention on intermediate or surrogate endpoints (see Section 2.3 below), the choice of the primary efficacy endpoint is particularly critical. A positive result may be misleading if the endpoint is not intrinsically involved in carcinogenic progression, but a negative result may lead to apparent trial failure despite underlying biological activity. Similar issues of biologic heterogeneity and clonal evolution complicate the development of cancer treatments, although they are magnified in preventive agent development due to the difficulties in tissue sampling and prediction of events and abnormalities that have not yet become biologically apparent.

### 2.2. Risk and Benefit Constraints

Cancer prevention trials have to operate within a narrower risk versus benefit window in comparison to treatment-focused trials, as they enroll largely healthy or asymptomatic individuals who may be exposed to long-term interventions and who may not develop cancer during the duration of the trial, or ever. In this setting, even modest toxicities can be deemed unacceptable when the potential benefit is a reduction in future cancer risk for only a subset of the exposed individuals rather than control of an active disease in the majority. While frequent but not life-threatening adverse events (e.g., low-grade diarrhea or nausea) limit participant compliance with long-term treatment, rare but serious events become unacceptable in the prevention setting.

The Adenoma Prevention with Celecoxib (APC) and Adenomatous Polyp Prevention on Vioxx (APPROVe) trials reflect this challenge. Although celecoxib and rofecoxib (Vioxx) significantly reduced colorectal adenoma recurrence, both were associated with dose-related increases in serious cardiovascular events [9,10,11]. The adverse events from the APPROVE and other trials ultimately led to market withdrawal of rofecoxib, and, similarly, the risk-benefit balance of celecoxib for cancer prevention was not deemed acceptable for long-term use for cancer prevention [12].

Similarly, in prostate cancer, the Prostate Cancer Prevention Trial (PCPT) showed that finasteride reduced overall cancer incidence but raised concerns for high-grade disease and caused sexual dysfunction [13]. Collectively, these examples highlight how a relatively small increase in serious adverse outcomes can outweigh the potential benefit of reducing cancer incidence.

An example of how tolerability restricts prevention strategies is illustrated by the breast cancer prevention trials, such as NSABP P-1 [14]. While tamoxifen significantly reduced invasive breast cancer in high-risk women, real-world use has remained modest due to a low but real absolute risk of endometrial cancer (albeit without increasing mortality), venous thromboembolism, and menopausal symptoms associated with treatment [15]. These challenges highlight that, unlike the treatment setting where patients and physicians may accept substantial toxicity for disease control, prevention agents must offer both risk reduction and a benign toxicity profile for acceptance by otherwise healthy individuals. The same agent used for different indications has considerably lower acceptance in the prevention setting.

Furthermore, there are no standard criteria for acceptable toxicity thresholds across cancer prevention settings. Instead, tolerability depends on baseline risk, anticipated benefit, and treatment duration, with evidence derived from individual trials rather than systematic cross-cancer comparisons [16,17]. Patient-reported outcomes (PROs) and adherence data help bridge this gap by providing insight into how patients experience preventive interventions. However, these measures remain underused as decision drivers in prevention trials, which complicates risk-benefit assessment [18].

Within prevention settings, low-grade symptoms, even if clinically mild, can disproportionately affect quality of life in asymptomatic populations and impact long-term adherence [19]. Because clinician-reported adverse events may underestimate symptom burden, PROs help capture symptoms that influence adherence [20]. Inconsistent adherence complicates efficacy assessment, as reduced drug exposure can mask treatment benefits, making it difficult to distinguish biological inefficacy from inadequate exposure [21]. Despite their relevance, PROs and adherence metrics are rarely used as primary or key secondary endpoints in early-phase cancer prevention trials. Challenges in standardization and lack of consensus thresholds limit their influence on trial design and regulatory use [22].

### 2.3. Measuring Preventive Efficacy

Assessing efficacy in cancer prevention trials is challenging because definitive (and approvable) endpoints such as cancer incidence and mortality occur years after an intervention begins and require large sample sizes for proper evaluation [23]. Unlike treatment settings, where response can be measured with CT or other imaging, these tools rarely detect the early biological effects of preventive agents, which occur at the level of dysplasia or molecular alterations that are not captured reliably by imaging [24]. As a result, many trials rely on intermediate or surrogate endpoints such as lesion regression, histologic grade, or biomarker modulation, even though the extent to which these measures reflect true cancer risk often remains uncertain. To be a good surrogate, a marker should be intrinsic to the process of carcinogenesis and to the mechanism of action of the intervention, such that modulating marker expression would also affect the course of the disease. Marker expression should differ between normal and diseased or at-risk epithelium, and measurements should be reproducible. To be a true surrogate, validation is critical [25,26]. Similar challenges are seen in cancer screening, where surrogate endpoints can shorten trial duration but require extensive validation to ensure they truly reflect true reductions in cancer incidence or mortality [27].

Thus far, no true surrogates have been validated for cancer prevention. Biomarkers assessed in most early-phase prevention trials remain exploratory, although they can be informative and help develop the body of evidence needed for subsequent phase III trials. Premalignant lesions are often used as endpoints even though their natural history remains highly variable. Oral leukoplakia, for example, may regress, persist, or progress to cancer, and reported malignant transformation rates have varied across studies. This variability complicates efforts to interpret changes in lesion size or dysplasia grade as definitive evidence of prevention [28]. Similar challenges arise elsewhere within the aerodigestive tract, where dysplasia endpoints in bronchial or esophageal prevention trials fluctuate over time and may improve without altering long-term cancer risk [5]. These uncertainties demonstrate that intermediate changes in histology or biomarkers do not reliably predict whether an intervention reduces cancer incidence and generally cannot be used as approvable endpoints in phase III trials, although they can be indicators of preliminary efficacy that subsequently need to be confirmed in larger phase III studies with cancer incidence endpoints [29]. Similarly, molecular endpoints such as methylation signatures, signaling pathway modulation, or immune profiling can be highly informative but are not sufficient for regulatory approval.

Evaluating endpoints also frequently requires tissue, which introduces additional limitations. Many cancer prevention studies depend on serial biopsies of premalignant tissue to assess histologic or molecular changes, leading to repeated invasive procedures over months or years. This requirement contributes to slow accrual, lower retention, and added procedural risk in otherwise healthy participants, and it limits the generalizability of trial results. Reliance on invasive endpoints is one of the key barriers to efficient chemoprevention research [23]. Although emerging noninvasive biomarkers, including circulating DNA assays and methylation-based signatures, are under investigation, they remain unvalidated and have not yet replaced tissue-based measures in most prevention settings.

### 2.4. Cohort Identification

Cohort selection is another difficult aspect of prevention trial design because very high-risk groups are not necessarily the most generalizable populations. High-risk hereditary syndromes such as *BRCA*1/2, Lynch, or Li-Fraumeni allow cancer endpoints to be reached sooner and can justify exposure to preventive agents. Where mechanisms of carcinogenesis are shared between high-risk (e.g., genetic) cohorts and individuals with sporadic cancers, trials initially in high-risk cohorts can offer a shortcut to drug development. However, in most cases, the mechanisms are not adequately understood and thus cannot be generalized to the population at large. The Metformin in Li-Fraumeni Syndrome (MILI) trial, for example, randomizes adults with Li-Fraumeni syndrome to metformin plus MRI surveillance versus surveillance alone, using five-year cancer-free survival as the primary endpoint. This design illustrates how very high-risk cohorts make event-driven prevention trials feasible and can justify long-term exposure to an oral agent that is regarded as safe but is not without any toxicities [29]. Should this trial be positive, it would be a major step forward for the care of these individuals who are at high risk for multiple different cancers, although it would not be generalizable to other populations since the rationale is partially based on the enhanced oxidative metabolism in p53-deficient cells from Li-Fraumeni patients and pertinent mouse models.

Equity and access limitations pose an additional challenge for cohort selection. Genomic-driven prevention trials offer biological precision, but access to genetic testing and specialized surveillance remains uneven. This raises concerns that such approaches could widen existing health disparities among socioeconomic, racial, and geographic groups [30]. Prevention strategies are often developed case-by-case, tailored to specific risks or molecular drivers, rather than designed for population-wide application [31]. While a targeted approach is necessary to demonstrate initial efficacy, extending these strategies to broader populations remains difficult, especially where genomic testing may be unavailable or inconsistently implemented [32].

Additionally, the identification of cohorts with premalignant lesions introduces a different set of selection challenges. Studies in these cohorts allow earlier, lesion-based endpoints, but these lesions may be relatively uncommon and show substantial variability within the underlying biology. Trials tend to accrue slowly due to the need to use invasive methods (e.g., colonoscopy or bronchoscopy, etc.) to screen multiple individuals to identify those who harbor the requisite lesions. These factors also limit their ability to represent broader at-risk populations. At the other end of the spectrum, trials in more broadly defined high-risk populations, such as postmenopausal women with elevated breast cancer risk, yield results that are more generalizable if they reach a cancer incidence endpoint, but this requires much larger sample sizes and longer follow-up to observe cancer events (e.g., phase III trial designs). The MAP.3 and IBIS-II trials, for instance, enrolled thousands of women at moderately increased risk and showed that exemestane and anastrozole, respectively, can reduce breast cancer incidence, but only with prolonged treatment and extended post-treatment follow-up [33,34].

Taken together, cancer prevention trials are limited by heterogeneous biology, narrow toxicity margins, unvalidated or invasive endpoints, and challenges in selecting appropriate cohorts. High genomic risk or lesion-based cohorts allow early endpoints but limit generalizability, whereas broader high-risk groups improve applicability at the cost of larger and longer trials. These constraints underscore the need for strategies that improve target selection, reduce toxicity, and make trial designs more feasible.

## 3. Advances in Prevention Trials: Transition to Precision Prevention

### 3.1. Overview of Precision Prevention

**Figure 1:**
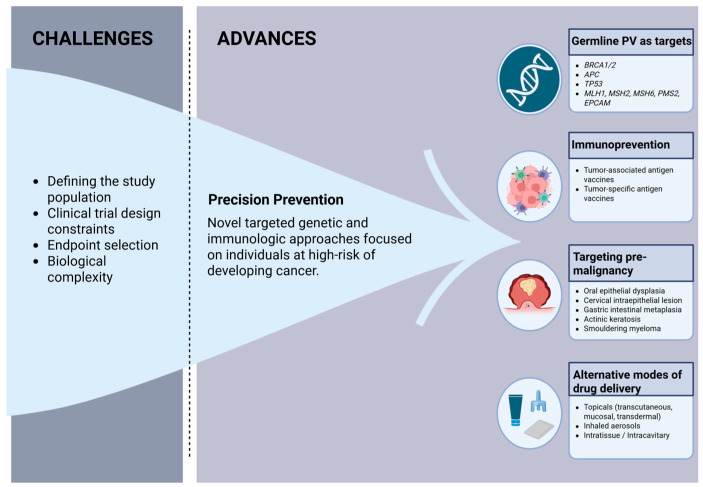
Challenges and advances in cancer prevention clinical trials. Created in BioRender. Francis, E. (2025) https://biorender.com/esurtm3 PV = pathogenic variant (Accessed on 12 December 2025).

Advances in genomics and digital health [35], together with the recognition of the need to refine cancer prevention trial endpoints [16], are prompting a shift from traditional prevention trials to precision prevention studies, which use targeted genetic and immunologic approaches in individuals at high risk of developing cancer to halt or “intercept” the progression from premalignant to invasive cancer (Figure 1) [36]. Contrary to traditional phase III prevention trials, which focused on population-level risk factors such as diet and smoking and frequently used repurposed interventions or diet-derived agents [37,38,39], precision prevention focuses on individualized risk prediction and novel biologically informed interventions in a smaller high-risk population [40]. Precision studies utilize short-term surrogate endpoints such as cancer incidence (in extremely high-risk populations) and biomarker changes and favor an integrated approach with treatment or surveillance protocols [40]. In this way, precision prevention studies will allow for a more targeted and mechanistically informed approach that is more likely to be cost-effective and faster to conduct than traditional prevention trials, which on average require 10–15 years to complete [40].

Though precision prevention trials allow for more targeted and efficient interventions, there are certain limitations to consider. For instance, over-stratification and small sample sizes diminish statistical power and increase variability, necessitating multi-center designs and adaptive analytics. Moreover, as surrogate endpoints may not reliably predict long-term reductions in disease risk, incorporating post-trial surveillance and leveraging population-level registry data with linked tissue resources [41] are important considerations. Given this, precision prevention trials complement rather than replace population-level prevention trials, which offer greater statistical stability and generalizability and benefit from established post-trial surveillance infrastructure.

### 3.2. Germline Pathogenic Variants as Therapeutic Targets

Germline pathogenic variants (PV) are heritable DNA changes present in all cells that predispose an individual to develop certain diseases, including cancer [42]. Well-established oncologic syndromes with associated germline PVs include hereditary breast and ovarian cancer (*BRCA*1/2), Lynch syndrome (*MLH1*, *MSH2*, *MSH6*, *PMS2*, and *EPCAM*), Li-Fraumeni syndrome (*TP53*), and Cowden syndrome (*PTEN*). Recent studies have focused on therapeutically targeting these germline PVs, fundamentally changing the management of certain cancer predisposition syndromes [8].

The use of PARP inhibitors (PARPi) in hereditary breast and ovarian cancer (HBOC) is the first key example of a germline PV as therapeutically actionable [43]. Germline and somatic mutations in *BRCA*1/2 lead to an inability to perform homologous repair of DNA defects [44]. PARPi, like olaparib, capitalize on this weakness as they block the repair of single-strand DNA breaks by inhibiting PARP enzymes on DNA, causing replication-associated double-strand breaks that are lethal to cells lacking functional homologous recombination [45]. In the late 2000s, the synthetic lethality of PARPi was confirmed in humans with advanced *BRCA*1/2-mutated advanced breast, ovarian, and prostate cancers [46]. This led to PARPi use for maintenance therapy in *BRCA*-mutated and homologous recombinant-deficient ovarian cancer [47,48,49] and expanded use of PARPi from the metastatic breast cancer setting [50,51] to early-stage germline *BRCA* breast cancer [50,52], where a significant improvement in overall survival was observed [53]. Though PARPis are not approved for the prevention of cancer in individuals with *BRCA*1/2 germline PV, this is an active area of investigation [54]. The development of next-generation PARP1-selective inhibitors, such as saruparib, offers the potential for more tolerable agents that may be better suited for cancer prevention [55].

When considering a drug for cancer prevention in such high-risk populations, several factors must be assessed, including the penetrance of the germline pathogenic variant, the drug’s tolerability and late effects, and the response of the agent in patients with germline PV who have developed cancer [56]. Using HBOC and PARPi again as an example, the penetrance of *BRCA*1/2 is high (68–80% for *BRCA*1 and 75–88% for *BRCA*2 for breast cancer; 60–65% for *BRCA*1 and 30–37% for *BRCA*2 for ovarian cancer) [57], and PARPi are well tolerated, albeit not without any side effects, as evidenced by a relatively low (<12%) permanent discontinuation of PARPi due to toxicity in pivotal maintenance trials [50,58]. Although our understanding of PARPi late effects is still evolving, the significant impact of PARPi on the management of early-stage and metastatic *BRCA*1/2 breast and ovarian cancer makes PARPi a potential agent for continued investigation for precision prevention in populations at very high cancer risk.

Though not reviewed in detail here, it is also important to recognize prevention trials pertaining to therapeutic agents in other cancer predisposition syndromes (Table 1). Due to their high cancer risk, individuals with cancer predisposition syndromes are frequently studied early during preventive drug development to enrich for the cancer incidence endpoint, as long as the underlying mechanisms are potentially similar to those giving rise to sporadic carcinogenesis. Thus, the trials in Table 1 do not necessarily reflect targeting the abnormal predisposition gene.

In contrast, many germline PVs (e.g., *APC I1307 K* and *ATM*) confer a modest increase in cancer risk and often act in concert with other genetic and environmental factors, making it difficult to justify the initiation of preventive therapeutics in these populations. A polygenic risk score (PRS) offers a quantitative measure of an individual’s risk for a disease [71,72,73], providing insight on which individuals may benefit from early preventive intervention. However, PRS determination has multiple limitations, including uncertain clinical thresholds, variable accuracy across populations, nonstandardized analytic approaches, and lack of prospective validation, constraining PRS use in prevention trials [74,75]. Though PRS shows promise as a personalized precision prevention strategy, providing insight not only on who is at greatest risk for disease development but also possibly guiding when and how intense to initiate therapy, more rigorous refinement and standardization are needed.

### 3.3. Immunoprevention

Since the 1980s, the field of immunoprevention, or the use of vaccines or immune-modulating strategies to prevent cancer [76,77], has enveloped three main phases—viral vaccines, antigen-based vaccines, and precision immunoprevention [36,63]. Vaccines against oncogenic viruses such as the human papillomavirus and hepatitis B virus [64] significantly reduced the incidence of cervical cancer and hepatocellular carcinoma, respectively [78,79,80,81]. Building on this success, the next phase of immunoprevention centers on antigen-based approaches, which established the groundwork for precision immunoprevention.

Antigen-based vaccines target either tumor-associated antigens (TAA), non-mutated self-proteins expressed in tumors, or tumor-specific antigens (TSA), typically mutation-derived neoantigens expressed in tumor cells [82]. Though they have had limited success in treating advanced cancers [82,83,84,85,86], these vaccines show promise in primary and secondary prevention (Table 2), which is our focus herein, and variable success in tertiary prevention and as adjuvant treatments [87,88,89]. Success in these settings is attributed to low disease burden [88,89], limited clonal heterogeneity, and a potentially less suppressed immune system [90,91]. Additional strategies employed by successful preventive antigen-based vaccines include concomitant administration of additional immunomodulatory drugs to overcome immunosuppression, careful consideration of the selected target antigen, and focus on high-risk populations such as those with premalignant lesions or germline pathologic variants (Table 2). For instance, the presence of the transmembrane glycoprotein mucin 1 (MUC1) in premalignant lesions as well as in several cancers such as colon and lung, Refs. [92,93] has made it a desirable TAA vaccine target, with promising results in the preclinical [94,95] and early clinical settings [96,97,98] in healthy individuals at risk of cancer development. Similarly, given the role of cancer stem cells (CSC) in cancer onset and metastasis [99,100], CSC antigens such as crypto-1 and cystine-glutamate antiporter xCT [101,102], which are expressed in early and invasive breast cancer, have emerged as compelling TAA vaccine targets that have shown promising anti-metastatic effects in preclinical analyses [102,103,104,105].

Tumor neoantigens, highly immunogenic tumor-specific proteins not expressed on normal cells [112], represents another promising target for prevention and forms the basis for TSA vaccines [113]. The possible success of TSAs as cancer prevention vaccines is predicated on utilizing neoantigens with high diversity, such as those formed by frameshift mutations [114] and gene fusions [115], rather than single-nucleotide variations, as the former produce a higher immunogenic response. Utilizing neoantigens that are less individualized, such as those from common oncogenic driver mutations like *KRAS*, *TP53*, and *BRAF*, allows for large-scale, cost-effective vaccine production [82] and could possibly prevent tumor evolution with antigen-loss variants [116]. For instance, a novel Lynch syndrome frameshift peptide vaccine is a first-in-kind precision preventive vaccine that targets neoantigens from recurrent microsatellite mutations common in Lynch syndrome patients, leveraging the active immune microenvironment of Lynch polyps associated with colorectal cancer [117,118]. Preclinical studies demonstrated robust immunogenic responses, reduced intestinal tumor burden, and prolonged overall survival in mice [119,120], providing strong support that neoantigen vaccines may be effective in Lynch syndrome and prompting initiation of an early phase clinical trial, which is ongoing, but has already confirmed feasibility, tolerance, and immunogenicity (NCT05078866) [110,111,121]. In addition to assessing late effects and long-term safety, future research will focus on confirming whether this vaccine does prevent Lynch-associated cancers.

Though a form of antigenic vaccines, TSAs reflect a broader transition in immunoprevention clinical trials to precision interception, an evolution made possible due to advances in sequencing and multi-omics [113] and a greater understanding of immunotherapy. It remains to be seen if TSA vaccines will outperform TAAs, but their role as cancer preventive agents remains an active area of investigation. Further, although the late effects of these vaccines remain unknown, they are currently administered to populations with a history of cancer or at high risk for developing a primary malignancy or recurrence. An extended follow-up will be required before considering their use in the general population at average cancer risk.

### 3.4. Targeting Known Premalignancy

Focusing on premalignant lesions in an attempt to prevent invasive cancer is not a new concept [122,123,124,125]. However, what has shifted is the framing of premalignant lesions as a targetable disease state with biologically defined risk, which can be modulated via multiple advanced modalities that consider molecular and immunological principles. For instance, the risk of progression from a premalignant state to invasive cancer can be quantified by considering multiple factors, including genomic characteristics [126], the immune microenvironment [127,128] and pathological features [129] such as *TP53* loss, aneuploidy, PD-L1 expression, and histological grade, as well as the presence of biomarkers like ctDNA [130] and targetable mutations such as *BRCA*1/2 [131]. Currently, only clinical and pathological features are used for clinical decisions; however, there is strong biological support to pursue the development of a multimodal risk system that incorporates other factors.

Artificial intelligence (AI) has primarily used single-modality data to risk-stratify premalignant lesions. For instance, AI deep learning has been applied to assess the histopathology of biopsied precursor lesions [132] and to non-invasively infer biological aggressiveness from imaging phenotypes [133,134]. Emerging multimodal AI approaches now integrate diverse data types to enhance predictive accuracy. Indeed, tissue-specific models such as TissueCypher, a multi-institutionally validated fluorescence imaging platform which uses AI to integrate various factors, including biomarkers (e.g., loss of tumor suppressor genes, amplification of oncogenes, alterations in lipid metabolism) [135,136] and tissue morphology, provides a 5-year risk score predictive of progression from nondysplastic or low-grade dysplastic Barrett’s esophagus to high-grade dysplasia or esophageal adenocarcinoma [129,137,138,139,140]. TissueCypher has superior risk stratification predictability than traditional approaches and has exhibited a meaningful impact on clinical management, affecting 55% of cases in a small prospective observational study [141]. While this represents a single, tissue-specific example, broader generalizations across cancers and tissue types remain challenging.

Efforts to understand and map out the multidimensional nature of multiple premalignant lesions have been the focus of the PreCancer Atlas (PCA) project [142], an initiative launched in 2018 as part of the Cancer Moonshot’s Human Tumor Atlas Network (HTAN) [143,144]. As of November 2025, HTAN teams have constructed 14 atlases using data from 20 organs and 64 cancer types. Precancer atlases have been reported on sporadic colorectal cancer [145,146,147], familial adenomatous polyposis-related colorectal cancer [148,149,150], lung cancer [151,152,153], breast cancer [154,155,156,157], melanoma [158,159], and pancreatic cancer [160]. By integrating multi-omic features such as genomic, transcriptomic, epigenetic, and immune microenvironmental profiles with established histological characteristics, PCA aims to provide clarity on why some premalignant lesions progress to invasive cancer, whereas others stabilize or regress. This multidimensional view may permit a more accurate risk stratification of multiple premalignant lesions, which can be utilized to develop targeted precision-prevention and early interception strategies [142,161].

### 3.5. Alternative Modes of Drug Delivery

Toxicity, including immediate adverse events and late effects, is critical to consider when considering an agent for cancer prevention, as discussed above, and tolerability and toxicity are major challenges to the long-term delivery of preventive interventions. Alternative dosing strategies are one approach to decreasing toxicity that has shown some success. For example, exemestane administration three times weekly was found to be noninferior to once daily dosing in a short window-of-opportunity pre-surgical trial, providing an approved agent as an alternative option for early-stage estrogen receptor-positive breast cancer patients intolerant to once daily dosing and providing the rationale for a larger, longer trial to assess breast cancer preventive efficacy [162]. Similarly, topical 5-fluorouracil (5-FU) was noted to have improved tolerance with biweekly dosing rather than with daily dosing or decreased concentrations [163,164,165].

**Figure 2:**
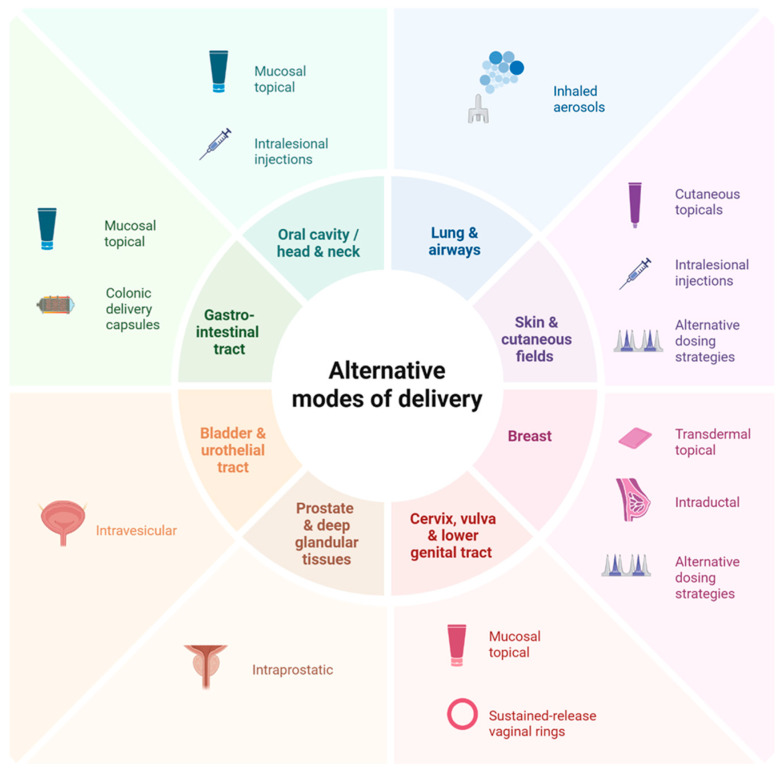
Alternative drug delivery strategies in cancer prevention. This figure highlights delivery modalities spanning the developmental spectrum, from those with robust preclinical evidence to those with established preventive use or those used in the treatment setting with emerging preventive potential. Created in BioRender. Francis, E. (2025) https://BioRender.com/p2prbz1 (Accessed on 12 December 2025).

Another approach to limit toxicity focuses on local organ-specific delivery rather than traditional systemic therapy (Figure 2) [166]. Regional drug delivery modalities include topical formulations, inhaled aerosols, and intra-tissue or intracavity approaches. Of these, topical agents (cutaneous, transdermal, and mucosal) are the most studied local preventive delivery modalities, for instance:

1. Cutaneous topicals: Cutaneous cancer-prevention topicals leverage direct delivery to the skin for local action. Approved monotherapy topical treatments such as 5-FU and imiquimod, and the investigational combination of calcipotriol with 5-FU, have shown good clearance of actinic keratosis (AK), the precursor lesion to squamous cell carcinoma (SCC) of the skin [167,168]. Indeed, the combination of calcipotriol with 5-FU was noted to significantly lower the risk of SCC development within three years of treatment and produced a robust T-cell response [168], prompting investigation of its use in high-risk groups with AK, such as transplant recipients (NCT05699603) [169].
2. Transdermal topicals: Transdermal topicals utilize the skin for systemic or loco-regional delivery of a drug, bypassing hepatic first-pass metabolism [170]. The transdermal tamoxifen metabolite 4-hydroxytamoxifen, delivered in gel form, was noted to have fewer systemic symptoms than oral tamoxifen, including reduced coagulation parameters, in patients with estrogen receptor-positive ductal carcinoma in situ of the breast, but has had mixed results on efficacy. One early randomized controlled trial noted that the gel matched the antiproliferative effect of oral tamoxifen [171], although this was not confirmed in a larger randomized trial by Khan et al., who suggested that future trials enhance metabolite delivery or use a more potent analog to improve response [172].
3. Mucosal topicals: Mucosal topicals have been studied across various anatomical locations, including the cervix [164,171,173,174], vagina [175,176], vulva [177,178], anus [179], oral cavity [180,181], and the gastrointestinal tract. Of these, agents targeting vulvar intraepithelial neoplasia (VIN) and cervical intraepithelial neoplasia (CIN), precursors to vulvar and cervical carcinoma, respectively, have shown the most promise. For instance, imiquimod has been associated with high durable response rates for VIN [182] and has been effective at treating high-grade CIN [183]. Though not superior to surgical resection, imiquimod is estimated to prevent surgery in at least 40% of high-grade CIN cases and may represent a non-surgical option that mitigates the toxicities associated with surgery [183].

A promising emerging use for preventive topical mucosal agents is in the gastrointestinal tract, with the development of hydrogels containing thiolated mucoadhesive polymers, which create temporary bonds with mucosal surfaces, extending drug contact time [184,185]. When combined with stimulus-release hydrogel technology, such as pH or enzyme-sensitive activation, including azo-polymeric hydrogels [186] and nanoparticle systems [187], thiolated hydrogels have the potential for controlled release of a drug specifically within the colonic mucosa [188,189]. To date, no trials have examined thiolated hydrogels as a primary chemoprevention strategy for colorectal cancer; however, pre-clinical studies have shown promise [190].

Beyond topical agents, other regional drug delivery modalities are emerging. For instance, inhalable aerosols, which take advantage of the lungs’ large surface area, thin epithelial barrier, and high vascularization [191] to directly deliver drugs in the airway, are an approved drug modality used to treat multiple pulmonary conditions, such as asthma. Given their ability to directly deliver drugs to premalignant lung lesions, their use as a preventive drug modality is currently under exploration. Multiple preclinical studies have noted significant reductions in pulmonary tumor multiplicity, load, and formation with various aerosolized agents [192,193,194,195]; however, in early-phase clinical trials, aerosols have shown modest effects [196,197]. While not yet demonstrating clear preventive benefit, the aerosolized delivery approach nevertheless remains an intriguing modality under active investigation and highlights a point of intersection between therapeutic advancements and potential preventive application. Similarly, intra-tissue or intracavity approaches (e.g., intravesical, intraductal, intraprostatic, intralesional injections, colonic delivery capsules, and vaginal rings) (NCT06623110) [188,198,199,200,201,202,203,204,205] also aim to directly deliver drugs to the organ systems of concern, and are emerging preventive modalities that will be watched closely. As with aerosols, many of these modalities originate from approved therapeutic uses in these organ systems.

## 4. Conclusions

With a rapidly increasing understanding of the biology of early carcinogenesis, cancer prevention is entering a new precision phase that promises to match the right intervention to the right individual. The past decade has seen major improvements in prevention target selection based on a deeper appreciation of premalignant biology at the epithelial as well as microenvironment and systemic immunity levels. The focus on homogeneous high-risk populations, such as those with germline mutations, enhances the likelihood of picking up an efficacy signal in early-phase clinical trials, although generalizability to the population at large remains to be determined. However, the possibility that many (maybe even most) preventive interventions will be limited to high-risk populations rather than average-risk individuals must be considered. Alternative approaches, such as screening or lifestyle interventions, may be needed for average-risk individuals.

The recognition of tremendous biological heterogeneity in tumor evolution, both between and within individuals, has reframed the approach to prevention in favor of targeting carcinogenic pathways over individual molecular abnormalities. Harnessing the immune system to eliminate premalignancy and prevent progression to invasive disease is an attractive approach being investigated in multiple cancer prevention settings. Additionally, attention to the delivery of effective therapies to optimize high compliance has resulted in multiple trials of localized drug delivery systems to optimize the risk-benefit calculation.

One of the largest challenges in cancer prevention is the uncertainty of who will develop cancer, when it will occur, and in which organ it will originate. However, individuals at high risk for one cancer remain at risk for other cancers and for other chronic diseases associated with similar exposures or aging. For instance, while heavy smoking is a risk factor for multiple tobacco-associated malignancies, an individual with such exposure still remains at risk for common non-tobacco-associated malignancies such as breast or prostate cancer. Approaches that target multiple cancer types by virtue of shared pathogenesis or shared molecular abnormalities, such as a MUC1 vaccine discussed above, are therefore very appealing, especially as more potent vaccines targeting multiple shared antigens are developed. Furthermore, individuals at risk for cancer are also at risk for multiple chronic diseases (e.g., heart disease and chronic obstructive pulmonary disease from heavy smoking), so approaches that target shared pathogenic mechanisms are particularly appealing [206]. It will take a multi-pronged effort addressing targets, cohort identification, trial design, optimal intervention delivery, and outcome assessment to make sustained progress, but recent advances make such progress within reach. Nevertheless, it must be recognized that preventive interventions cannot exist in a vacuum and must be integrated with population-level strategies such as lifestyle interventions (e.g., tobacco and alcohol cessation, obesity control) and appropriate screening strategies to optimally decrease cancer incidence.

## Figures and Tables

**Table 1:** Clinical trials in individuals with germline pathogenic variants (PV).

Cancer Predisposition Syndrome	Study	Population	Intervention	Putative Mechanism of Action	Design	Primary Endpoint(s)	Key Finding(s)
Hereditary breast and ovarian cancer (*BRCA*1/2)	*BRCA*-P Trial (NCT04711109) [59]	Women with germline *BRCA*1 PV	Denosumab versus placebo	Inhibition of RANKL and PR signaling in *BRCA*1 progenitors [60,61]	Phase III	Breast cancer risk reduction	Ongoing. Anticipate primary completion in July 2027 and study completion in December 2033.
Lynch syndrome (pathogenic mismatch repair including *MLH1*, *MSH2*, *MSH6*, *PMS2*, or *EPCAM*)	CaPP2 (ISRCTN59521990) [62]	Lynch syndrome carriers or high-risk relatives with prior cured neoplasm and intact colon	Aspirin 600 mg daily versus placebo	Mechanisms unclear (COX inhibition, possibly others)	Randomized	Development of colorectal cancer (size, number, and stage after two years of aspirin)	Over the 10-year follow-up, the incidence of colorectal cancer was less in participants who received aspirin versus placebo, 9% versus 13%, respectively.
	CaPP3 (NCT02497820)	Lynch syndrome carriers	Aspirin. Blinded daily dose (600 mg versus 300 mg versus 100 mg) followed by open label dose of 100 mg daily	Mechanisms unclear (COX inhibition, possibly others)	Phase III, Randomized	Dose- dependent cancer prevention after ≥ 2 years of treatment via a comparison of cancer incidence rates after 5 years	Results pending publication. Per press releases in June 2025, CaPP3 has noted that lower doses of aspirin (100 mg daily) are as effective as higher doses at decreasing colorectal cancer incidence in Lynch syndrome carriers [63,64].
Familial adenomatous polyposis (*APC*)	NCT01483144 [65,66,67]	Adults with familial adenomatous polyposis (FAP) and polyps	Combination eflornithine + sulindac versus single-agent sulindac or single-agent eflornithine	Inhibition of ODC and polyamine synthesis (eflornithine) and COX inhibition, possibly others (sulindac)	Phase III, Randomized	Disease progression requiring polyp excision or major surgery or high-grade dysplasia	No significant reduction in disease progression was observed between the combination and single-agent arms [67]. However, post hoc analyses noted that combination therapy was superior to either single-agent at delaying or preventing lower GI surgery [65].
	Celecoxib in FAP Trial (No NCT) [68]	Adults with FAP and polyps	Celecoxib (100 mg or 400 mg twice daily) versus placebo	COX-2 inhibition, possibly others	Randomized	Adenoma regression at six months	Compared to placebo, celecoxib reduced the size and number of colorectal polyps, with a greater improvement observed in participants receiving celecoxib 400 mg twice daily.
	Sulindac in FAP Trial (No NCT) [69]	Adults with FAP with intact colons or subtotal colectomy or ≥5 adenomatous polyps	Sulindac versus placebo for 9 months	COX inhibition, possibly others	Randomized	Adenoma regression over 12 months, per endoscopic evaluation every three months	Compared to placebo, sulindac reduced the size and number of colorectal adenomas with the first notable and maximal effects at three and six months, respectively. However, polyp size and number increased at month 12 after terminating sulindac three months prior.
Li Fraumeni syndrome (*TP53*)	MILI Trial (ISRCTN16699730) [29]	Germline pathogenic variant of *TP53* with no active cancer	Metformin + annual MRI versus annual MRI surveillance	Mechanisms unclear (mitochondrial OXPHOS inhibition, others) [70]	Phase II, randomized	Cumulative cancer-free survival at 5 years	Ongoing. Anticipate recruitment completion in December 2025, final results in December 2030.

AMPK = 5′ adenosine monophosphate-activated protein kinase; COX = cyclooxygenase; ER = estrogen receptor; OXPHOS = Oxidative phosphorylation; PR = progesterone receptor; RANK = Receptor Activator of Nuclear factor Kappa-B; RANKL = Receptor Activator of Nuclear factor Kappa-B Ligand.

**Table 2:** Clinical trials examining antigen-based vaccines for primary and secondary prevention.

Study	Population	Antigen	Design	Primary Endpoint(s)	Key Finding(s)
Tumor Associated Antigen (TAA)-based Vaccines					
NCT007773097 [96,97]	Presence of high-risk colonic adenomas	MUC-1	Phase I/II	Safety and vaccine immunogenicity	The first TAA vaccine trial in healthy participants at risk for colon cancer. Demonstrates safety, immunogenicity, and elucidation of long-term memory. Noted that non-responders exhibited high levels of myeloid-derived suppressor cells (MDSC), creating an immunosuppressive environment.
NCT02134925 [98]	Presence of high-risk colonic adenomas	MUC-1	Phase II, randomized trial	Vaccine immunogenicity	The vaccine reduced adenoma recurrence in participants with an immune response to the vaccine, although an immune response occurred in only 25%. MDSC, IL-6, and IL-8 levels were significantly elevated in non-responders.
NCT03300817 [106]	Current and former heavy smokers	MUC-1	Phase I	Safety and vaccine immunogenicity (IgG anti-MUC1 antibody titer)	Low vaccine response and high circulating levels of MDSCs suggest that immunosuppression may limit response to preventive vaccines in heavy smokers.
NCT05419011	Lynch syndrome carriers	Three antigens [MUC-1, brachyury and carcino-embryonic antigen (CEA)] + IL-15 super agonist	Phase IIb	Incidence of colorectal neoplasms	Ongoing. Anticipate primary completion in July 2027 and study completion in January 2028.
NCT04367675 [107]	*BRCA*1/2 carriers without active cancer	DNA plasmid vaccine encoding three TAAs [human telomerase reverse transcriptase (hTERT), prostate specific membrane antigen (PSMA), Wilms tumor gene-1 (WT1)] +/− IL12	Phase Ib	Safety	Demonstrated safety with most common adverse event being injection site reactions.
Tumor Specific Antigen (TSA)-based Vaccines					
NCT05013216 [108,109]	High-risk of developing pancreatic ductal adenocarcinoma (PDAC) due to family history or germline mutation	Synthetic long peptides corresponding to six common mutant *KRAS* (m*KRAS*) subtypes	Phase I	Safety and T-cell response	Ongoing with study completion, anticipated in May 2031. To date, the vaccine has been noted to elicit an appropriate m*KRAS*-specific T cell response. Minimal adverse effects were noted from the vaccine (e.g., injection site reaction, fatigue, chills, and headaches).
NCT05078866 [110,111]	Lynch syndrome with no active cancer	Frameshift neoantigen vaccine consisting of 209 neoepitopes from recurrent microsatellite mutations common in Lynch, as identified by extensive tumor sequencing	Phase Ib/II	Safety and immunogenicity	Ongoing with study completion, anticipated in September 2026. To date, the vaccine has been well tolerated and is noted to produce robust immunogenicity.

## Data Availability

No new data were created or analyzed in this study.

## Abbreviations

The following abbreviations are used in this manuscript:

AK	Actinic keratosis
AMPK	5′ adenosine monophosphate-activated protein kinase
CIN	Cervical intraepithelial neoplasia
COX	Cyclooxygenase
CSC	Cancer stem cell
CT	Computed tomography
ER	Estrogen receptor
FAP	Familial adenomatous polyposis
5-FU	5-Fluorouracil
HBOC	Hereditary breast and ovarian cancer
MDSC MRI	Myeloid-derived suppressor cells Magnetic Resonance Imaging
OXPHOS	Oxidative phosphorylation
PARPi	Poly (ADP-ribose) Polymerase (PARP) inhibitor
PR	Progesterone receptor
PV	Pathogenic variant
RANK	Receptor Activator of Nuclear Factor Kappa-B
RANKL	Receptor Activator of Nuclear Factor Kappa-B Ligand
SCC	Squamous cell carcinoma
TAA	Tumor-associated antigen
TSA	Tumor-specific antigen
VIN	vulvar intraepithelial neoplasia

## References

[1] Bray F., Laversanne M., Sung H., Ferlay J., Siegel R.L., Soerjomataram I., Jemal A. (2024). Global cancer statistics 2022: GLOBOCAN estimates of incidence and mortality worldwide for 36 cancers in 185 countries. CA Cancer J. Clin..

[2] Rebbeck T.R., Burns-White K., Chan A.T., Emmons K., Freedman M., Hunter D.J., Kraft P., Laden F., Mucci L., Parmigiani G. (2018). Precision Prevention and Early Detection of Cancer: Fundamental Principles. Cancer Discov..

[3] Dakubo G.D., Jakupciak J.P., Birch-Machin M.A., Parr R.L. (2007). Clinical implications and utility of field cancerization. Cancer Cell Int..

[4] De Bruin E.C., McGranahan N., Mitter R., Salm M., Wedge D.C., Yates L., Jamal-Hanjani M., Shafi S., Murugaesu N., Rowan A.J. (2014). Spatial and temporal diversity in genomic instability processes defines lung cancer evolution. Science.

[5] Han S., Bommireddy R., Kim P., Selvaraj P., Shin D.M. (2024). Chemoprevention of Head and Neck Cancer: A Review of Current Approaches and Future Perspectives. Cancer Prev. Res..

[6] Willenbrink T.J., Ruiz E.S., Cornejo C.M., Schmults C.D., Arron S.T., Jambusaria-Pahlajani A. (2020). Field cancerization: Definition, epidemiology, risk factors, and outcomes. J. Am. Acad. Dermatol..

[7] Lomakin A., Svedlund J., Strell C., Gataric M., Shmatko A., Rukhovich G., Park J.S., Ju Y.S., Dentro S., Kleshchevnikov V. (2022). Spatial genomics maps the structure, nature and evolution of cancer clones. Nature.

[8] Tang T.-Y., Colbert Maresso K., Ngeow J., Vilar E., Yap T.A. (2025). Germline Mutations as Cancer Drug Targets. Cancer Discov..

[9] Baron J.A., Sandler R.S., Bresalier R.S., Quan H., Riddell R., Lanas A., Bolognese J.A., Oxenius B., Horgan K., Loftus S. (2006). A Randomized Trial of Rofecoxib for the Chemoprevention of Colorectal Adenomas. Gastroenterology.

[10] Bertagnolli M.M., Eagle C.J., Zauber A.G., Redston M., Solomon S.D., Kim K., Tang J., Rosenstein R.B., Wittes J., Corle D. (2006). Celecoxib for the Prevention of Sporadic Colorectal Adenomas. N. Engl. J. Med..

[11] Bresalier R.S., Sandler R.S., Quan H., Bolognese J.A., Oxenius B., Horgan K., Lines C., Riddell R., Morton D., Lanas A. (2005). Cardiovascular Events Associated with Rofecoxib in a Colorectal Adenoma Chemoprevention Trial. N. Engl. J. Med..

[12] Singh D. (2004). Merck withdraws arthritis drug worldwide. BMJ.

[13] Thompson I.M., Goodman P.J., Tangen C.M., Lucia M.S., Miller G.J., Ford L.G., Lieber M.M., Cespedes R.D., Atkins J.N., Lippman S.M. (2003). The Influence of Finasteride on the Development of Prostate Cancer. N. Engl. J. Med..

[14] Fisher B., Costantino J.P., Wickerham D.L., Redmond C.K., Kavanah M., Cronin W.M., Vogel V., Robidoux A., Dimitrov N., Atkins J. (1998). Tamoxifen for Prevention of Breast Cancer: Report of the National Surgical Adjuvant Breast and Bowel Project P-1 Study. JNCI J. Natl. Cancer Inst..

[15] Waters E.A., Cronin K.A., Graubard B.I., Han P.K., Freedman A.N. (2010). Prevalence of Tamoxifen Use for Breast Cancer Chemoprevention Among U.S. Women. Cancer Epidemiol. Biomarkers Prev..

[16] Szabo E. (2006). Selecting targets for cancer prevention: Where do we go from here?. Nat. Rev. Cancer.

[17] Freedman A.N., Yu B., Gail M.H., Costantino J.P., Graubard B.I., Vogel V.G., Anderson G.L., McCaskill-Stevens W. (2011). Benefit/Risk Assessment for Breast Cancer Chemoprevention with Raloxifene or Tamoxifen for Women Age 50 Years or Older. J. Clin. Oncol..

[18] Basch E., Reeve B.B., Mitchell S.A., Clauser S.B., Minasian L.M., Dueck A.C., Mendoza T.R., Hay J., Atkinson T.M., Abernethy A.P. (2014). Development of the National Cancer Institute’s Patient-Reported Outcomes Version of the Common Terminology Criteria for Adverse Events (PRO-CTCAE). JNCI J. Natl. Cancer Inst..

[19] Smith S.G., Sestak I., Howell A., Forbes J., Cuzick J. (2017). Participant-Reported Symptoms and Their Effect on Long-Term Adherence in the International Breast Cancer Intervention Study I (IBIS I). J. Clin. Oncol..

[20] Atkinson T.M., Ryan S.J., Bennett A.V., Stover A.M., Saracino R.M., Rogak L.J., Jewell S.T., Matsoukas K., Li Y., Basch E. (2016). The association between clinician-based common terminology criteria for adverse events (CTCAE) and patient-reported outcomes (PRO): A systematic review. Support. Care Cancer.

[21] Chlebowski R.T., Kim J., Haque R. (2014). Adherence to Endocrine Therapy in Breast Cancer Adjuvant and Prevention Settings. Cancer Prev. Res..

[22] Mitchell S.A., Kluetz P.G., Chingos D.T., Basch E.M. (2016). Patient-Reported Outcomes in Cancer Clinical Trials: Measuring Symptomatic Adverse Events with the National Cancer Institute’s Patient-Reported Outcomes Version of the Common Terminology Criteria for Adverse Events (PRO-CTCAE). Am. Soc. Clin. Oncol. Educ. Book.

[23] Enserro D.M., Gunn H.J., Elsaid M.I., Duan F., Pugh S.L. (2025). Challenges to and considerations of designing cancer prevention trials. JNCI Monogr..

[24] Ren J., Yan G., Yang L., Kong L., Guan Y., Sun H., Liu C., Liu L., Han Y., Wang X. (2025). Cancer chemoprevention: Signaling pathways and strategic approaches. Signal Transduct. Target. Ther..

[25] Schatzkin A., Gail M. (2002). The promise and peril of surrogate end points in cancer research. Nat. Rev. Cancer.

[26] Szabo E. (2008). Assessing efficacy in early-phase cancer prevention trials: The case of oral premalignancy. Cancer Prev. Res..

[27] Webb A.B., Berg C.D., Castle P.E., Crosby D., Etzioni R., Kessler L.G., Menon U., Parmar M., Steele R.J.C., Sasieni P.D. (2024). Considerations for using potential surrogate endpoints in cancer screening trials. Lancet Oncol..

[28] Palma V.D.M., Koerich Laureano N., Frank L.A., Rados P.V., Visioli F. (2023). Chemoprevention in oral leukoplakia: Challenges and current landscape. Front. Oral Health.

[29] Dixon-Zegeye M., Shaw R., Collins L., Perez-Smith K., Ooms A., Qiao M., Pantziarka P., Izatt L., Tischkowitz M., Harrison R.E. (2024). Cancer Precision-Prevention trial of Metformin in adults with Li Fraumeni syndrome (MILI) undergoing yearly MRI surveillance: A randomised controlled trial protocol. Trials.

[30] Manrai A.K., Funke B.H., Rehm H.L., Olesen M.S., Maron B.A., Szolovits P., Margulies D.M., Loscalzo J., Kohane I.S. (2016). Genetic Misdiagnoses and the Potential for Health Disparities. N. Engl. J. Med..

[31] Szabo E. (2008). Primer: First do no harm—When is it appropriate to plan a cancer prevention clinical trial?. Nat. Clin. Pract. Oncol..

[32] Roberts M.C., Dotson W.D., DeVore C.S., Bednar E.M., Bowen D.J., Ganiats T.G., Green R.F., Hurst G.M., Philp A.R., Ricker C.N. (2018). Delivery Of Cascade Screening For Hereditary Conditions: A Scoping Review Of The Literature. Health Aff..

[33] Cuzick J., Sestak I., Forbes J.F., Dowsett M., Knox J., Cawthorn S., Saunders C., Roche N., Mansel R.E., Von Minckwitz G. (2014). Anastrozole for prevention of breast cancer in high-risk postmenopausal women (IBIS-II): An international, double-blind, randomised placebo-controlled trial. Lancet.

[34] Goss P.E., Ingle J.N., Alés-Martínez J.E., Cheung A.M., Chlebowski R.T., Wactawski-Wende J., McTiernan A., Robbins J., Johnson K.C., Martin L.W. (2011). Exemestane for Breast-Cancer Prevention in Postmenopausal Women. N. Engl. J. Med..

[35] August G.J., Gewirtz A. (2019). Moving Toward a Precision-Based, Personalized Framework for Prevention Science: Introduction to the Special Issue. Prev. Sci..

[36] Stanton S.E., Castle P.E., Finn O.J., Sei S., Emens L.A. (2024). Advances and challenges in cancer immunoprevention and immune interception. J. Immunother. Cancer.

[37] Omenn G.S. (2000). Chemoprevention of Lung Cancer Is Proving Difficult and Frustrating, Requiring New Approaches. J. Natl. Cancer Inst..

[38] Omenn G.S., Goodman G.E., Thornquist M.D., Balmes J., Cullen M.R., Glass A., Keogh J.P., Meyskens F.L., Valanis B., Williams J.H. (1996). Risk factors for lung cancer and for intervention effects in CARET, the Beta-Carotene and Retinol Efficacy Trial. J. Natl. Cancer Inst..

[39] The ATBC cancer prevention study group (1994). The alpha-tocopherol, beta-carotene lung cancer prevention study: Design, methods, participant characteristics, and compliance. Ann. Epidemiol..

[40] Blagden S.P., Dodd K.W., Brown K., Szabo E. (2025). Precision Prevention Studies: A Targeted Approach to Cancer Prevention. Cancer Prev. Res..

[41] Sanchez P., Van Dyke A.L., Petkov V.I., Yuan Y., Bonds S., Valenzuela C., Tuan A.W., Moravec R., Altekruse S.F., Singhi A.D. (2024). NCI SEER-Linked Virtual Tissue Repository Pilot. J. Natl. Cancer Inst. Monogr..

[42] Rahman N. (2014). Realizing the promise of cancer predisposition genes. Nature.

[43] Bryant H.E., Schultz N., Thomas H.D., Parker K.M., Flower D., Lopez E., Kyle S., Meuth M., Curtin N.J., Helleday T. (2005). Specific killing of BRCA2-deficient tumours with inhibitors of poly(ADP-ribose) polymerase. Nature.

[44] Dziadkowiec K.N., Gąsiorowska E., Nowak-Markwitz E., Jankowska A. (2016). PARP inhibitors: Review of mechanisms of action and BRCA1/2 mutation targeting. Prz. Menopauzalny Menopause Rev..

[45] Helleday T. (2011). The underlying mechanism for the PARP and BRCA synthetic lethality: Clearing up the misunderstandings. Mol. Oncol..

[46] Fong P.C., Boss D.S., Yap T.A., Tutt A., Wu P., Mergui-Roelvink M., Mortimer P., Swaisland H., Lau A., O’Connor M.J. (2009). Inhibition of Poly(ADP-Ribose) Polymerase in Tumors from *BRCA* Mutation Carriers. N. Engl. J. Med..

[47] Coleman R.L., Oza A.M., Lorusso D., Aghajanian C., Oaknin A., Dean A., Colombo N., Weberpals J.I., Clamp A., Scambia G. (2017). Rucaparib maintenance treatment for recurrent ovarian carcinoma after response to platinum therapy (ARIEL3): A randomised, double-blind, placebo-controlled, phase 3 trial. Lancet.

[48] Ledermann J., Harter P., Gourley C., Friedlander M., Vergote I., Rustin G., Scott C., Meier W., Shapira-Frommer R., Safra T. (2012). Olaparib Maintenance Therapy in Platinum-Sensitive Relapsed Ovarian Cancer. N. Engl. J. Med..

[49] Moore K., Colombo N., Scambia G., Kim B.-G., Oaknin A., Friedlander M., Lisyanskaya A., Floquet A., Leary A., Sonke G.S. (2018). Maintenance Olaparib in Patients with Newly Diagnosed Advanced Ovarian Cancer. N. Engl. J. Med..

[50] Litton J.K., Rugo H.S., Ettl J., Hurvitz S.A., Gonçalves A., Lee K.-H., Fehrenbacher L., Yerushalmi R., Mina L.A., Martin M. (2018). Talazoparib in Patients with Advanced Breast Cancer and a Germline *BRCA* Mutation. N. Engl. J. Med..

[51] Robson M., Im S.-A., Senkus E., Xu B., Domchek S.M., Masuda N., Delaloge S., Li W., Tung N., Armstrong A. (2017). Olaparib for Metastatic Breast Cancer in Patients with a Germline *BRCA* Mutation. N. Engl. J. Med..

[52] Tutt A.N.J., Garber J.E., Kaufman B., Viale G., Fumagalli D., Rastogi P., Gelber R.D., De Azambuja E., Fielding A., Balmaña J. (2021). Adjuvant Olaparib for Patients with *BRCA1*—Or *BRCA2* -Mutated Breast Cancer. N. Engl. J. Med..

[53] Geyer C.E., Garber J.E., Gelber R.D., Yothers G., Taboada M., Ross L., Rastogi P., Cui K., Arahmani A., Aktan G. (2022). Overall survival in the OlympiA phase III trial of adjuvant olaparib in patients with germline pathogenic variants in BRCA1/2 and high-risk, early breast cancer. Ann. Oncol..

[54] To C., Kim E.-H., Royce D.B., Williams C.R., Collins R.M., Risingsong R., Sporn M.B., Liby K.T. (2014). The PARP inhibitors, veliparib and olaparib, are effective chemopreventive agents for delaying mammary tumor development in BRCA1-deficient mice. Cancer Prev. Res..

[55] Yap T.A., Im S.-A., Schram A.M., Sharp A., Balmana J., Baird R.D., Brown J.S., Schwaederle M., Pilling E.A., Moorthy G. (2022). Abstract CT007: PETRA: First in class, first in human trial of the next generation PARP1-selective inhibitor AZD5305 in patients (pts) with BRCA1/2, PALB2 or RAD51C/D mutations. Cancer Res..

[56] Pilié P.G., Gay C.M., Byers L.A., O’Connor M.J., Yap T.A. (2019). PARP Inhibitors: Extending Benefit Beyond BRCA-Mutant Cancers. Clin. Cancer Res..

[57] Evans D.G., Shenton A., Woodward E., Lalloo F., Howell A., Maher E.R. (2008). Penetrance estimates for BRCA1 and BRCA2 based on genetic testing in a Clinical Cancer Genetics service setting: Risks of breast/ovarian cancer quoted should reflect the cancer burden in the family. BMC Cancer.

[58] Colombo N., Moore K., Scambia G., Oaknin A., Friedlander M., Lisyanskaya A., Floquet A., Leary A., Sonke G.S., Gourley C. (2021). Tolerability of maintenance olaparib in newly diagnosed patients with advanced ovarian cancer and a BRCA mutation in the randomized phase III SOLO1 trial. Gynecol. Oncol..

[59] Bhulani N., Wood M., Tsai J., Bedrosian I., Hopkins J.O., Brunet J., Michaelson-Cohen R., Schmutzler R.K., Evans G.D., Gnant M. (2022). A phase 3 study to determine the breast cancer risk reducing effect of denosumab in women carrying a germline *BRCA1* mutation (BRCA-P Study). J. Clin. Oncol..

[60] Sigl V., Owusu-Boaitey K., Joshi P.A., Kavirayani A., Wirnsberger G., Novatchkova M., Kozieradzki I., Schramek D., Edokobi N., Hersl J. (2016). RANKL/RANK control *Brca1* mutation-driven mammary tumors. Cell Res..

[61] Nolan E., Vaillant F., Branstetter D., Pal B., Giner G., Whitehead L., Lok S.W., Mann G.B., Rohrbach K., Kathleen Cuningham Foundation Consortium for Research into Familial Breast Cancer (kConFab) (2016). RANK ligand as a potential target for breast cancer prevention in *BRCA1*-mutation carriers. Nat. Med..

[62] Iman A. (2025). Low-Dose Aspirin Can Prevent Bowel Cancer in People with Lynch Syndrome. Cancer Research UK. https://news.cancerresearchuk.org/2025/06/24/capp3-low-dose-of-aspirin-can-prevent-bowel-cancer-in-people-with-lynch-syndrome/.

[63] Haldar S.D., Vilar E., Maitra A., Zaidi N. (2023). Worth a Pound of Cure? Emerging Strategies and Challenges in Cancer Immunoprevention. Cancer Prev. Res..

[64] Beasley R.P., Lin C.-C., Hwang L.-Y., Chien C.-S. (1981). HEPATOCELLULAR CARCINOMA AND HEPATITIS B VIRUS. Lancet.

[65] Balaguer F., Stoffel E.M., Burke C.A., Dekker E., Samadder N.J., Van Cutsem E., Lynch P.M., Wise P.E., Hüneburg R., Lim R.M. (2022). Combination of Sulindac and Eflornithine Delays the Need for Lower Gastrointestinal Surgery in Patients with Familial Adenomatous Polyposis: Post Hoc Analysis of a Randomized Clinical Trial. Dis. Colon Rectum.

[66] Burke C.A., Dekker E., Samadder N.J., Stoffel E., Cohen A. (2016). Efficacy and safety of eflornithine (CPP-1X)/sulindac combination therapy versus each as monotherapy in patients with familial adenomatous polyposis (FAP): Design and rationale of a randomized, double-blind, Phase III trial. BMC Gastroenterol..

[67] Burke C.A., Dekker E., Lynch P., Samadder N.J., Balaguer F., Hüneburg R., Burn J., Castells A., Gallinger S., Lim R. (2020). Eflornithine plus Sulindac for Prevention of Progression in Familial Adenomatous Polyposis. N. Engl. J. Med..

[68] Steinbach G., Lynch P.M., Phillips R.K.S., Wallace M.H., Hawk E., Gordon G.B., Wakabayashi N., Saunders B., Shen Y., Fujimura T. (2000). The Effect of Celecoxib, a Cyclooxygenase-2 Inhibitor, in Familial Adenomatous Polyposis. N. Engl. J. Med..

[69] Giardiello F.M., Hamilton S.R., Krush A.J., Piantadosi S., Hylind L.M., Celano P., Booker S.V., Robinson C.R., Offerhaus G.J.A. (1993). Treatment of Colonic and Rectal Adenomas with Sulindac in Familial Adenomatous Polyposis. N. Engl. J. Med..

[70] Wang P.-Y., Li J., Walcott F.L., Kang J.-G., Starost M.F., Talagala S.L., Zhuang J., Park J.-H., Huffstutler R.D., Bryla C.M. (2017). Inhibiting mitochondrial respiration prevents cancer in a mouse model of Li-Fraumeni syndrome. J. Clin. Investig..

[71] Lewis C.M., Vassos E. (2020). Polygenic risk scores: From research tools to clinical instruments. Genome Med..

[72] Mavaddat N., Michailidou K., Dennis J., Lush M., Fachal L., Lee A., Tyrer J.P., Chen T.-H., Wang Q., Bolla M.K. (2019). Polygenic Risk Scores for Prediction of Breast Cancer and Breast Cancer Subtypes. Am. J. Hum. Genet..

[73] Wray N.R., Ripke S., Mattheisen M., Trzaskowski M., Byrne E.M., Abdellaoui A., Adams M.J., Agerbo E., Air T.M., Andlauer T.M.F. (2018). Genome-wide association analyses identify 44 risk variants and refine the genetic architecture of major depression. Nat. Genet..

[74] Ndong Sima C.A.A., Step K., Swart Y., Schurz H., Uren C., Möller M. (2024). Methodologies underpinning polygenic risk scores estimation: A comprehensive overview. Hum. Genet..

[75] Martínez-Minguet D., Noel R., Simón A.G., Pastor Ó. (2025). Challenges in clinical translation of polygenic risk score analyses: A systematic review. Genet. Med..

[76] Finn O.J., Beatty P.L. (2016). Cancer immunoprevention. Curr. Opin. Immunol..

[77] Roeser J.C., Leach S.D., McAllister F. (2015). Emerging strategies for cancer immunoprevention. Oncogene.

[78] Chang M.-H., Chen C.-J., Lai M.-S., Hsu H.-M., Wu T.-C., Kong M.-S., Liang D.-C., Shau W.-Y., Chen D.-S. (1997). Universal Hepatitis B Vaccination in Taiwan and the Incidence of Hepatocellular Carcinoma in Children. N. Engl. J. Med..

[79] Chang M.-H., You S.-L., Chen C.-J., Liu C.-J., Lee C.-M., Lin S.-M., Chu H.-C., Wu T.-C., Yang S.-S., Kuo H.-S. (2009). Decreased Incidence of Hepatocellular Carcinoma in Hepatitis B Vaccinees: A 20-Year Follow-up Study. JNCI J. Natl. Cancer Inst..

[80] Falcaro M., Soldan K., Ndlela B., Sasieni P. (2024). Effect of the HPV vaccination programme on incidence of cervical cancer and grade 3 cervical intraepithelial neoplasia by socioeconomic deprivation in England: Population based observational study. BMJ.

[81] Lei J., Ploner A., Elfström K.M., Wang J., Roth A., Fang F., Sundström K., Dillner J., Sparén P. (2020). HPV Vaccination and the Risk of Invasive Cervical Cancer. N. Engl. J. Med..

[82] Ruzzi F., Riccardo F., Conti L., Tarone L., Semprini M.S., Bolli E., Barutello G., Quaglino E., Lollini P.-L., Cavallo F. (2025). Cancer vaccines: Target antigens, vaccine platforms and preclinical models. Mol. Aspects Med..

[83] Bezu L., Kepp O., Cerrato G., Pol J., Fucikova J., Spisek R., Zitvogel L., Kroemer G., Galluzzi L. (2018). Trial watch: Peptide-based vaccines in anticancer therapy. OncoImmunology.

[84] Goydos J.S., Elder E., Whiteside T.L., Finn O.J., Lotze M.T. (1996). A Phase I Trial of a Synthetic Mucin Peptide Vaccine. J. Surg. Res..

[85] Li F., Deng L., Jackson K.R., Talukder A.H., Katailiha A.S., Bradley S.D., Zou Q., Chen C., Huo C., Chiu Y. (2021). Neoantigen vaccination induces clinical and immunologic responses in non-small cell lung cancer patients harboring EGFR mutations. J. Immunother. Cancer.

[86] Ott P.A., Hu-Lieskovan S., Chmielowski B., Govindan R., Naing A., Bhardwaj N., Margolin K., Awad M.M., Hellmann M.D., Lin J.J. (2020). A Phase Ib Trial of Personalized Neoantigen Therapy Plus Anti-PD-1 in Patients with Advanced Melanoma, Non-small Cell Lung Cancer, or Bladder Cancer. Cell.

[87] Hu L., Zhang Y., Kong X., Wu Z., Wang H. (2024). Impact of personalized peptide neoantigen vaccines on immunologic responses in patients with pancreatic cancer. J. Clin. Oncol..

[88] Ott P.A., Hu Z., Keskin D.B., Shukla S.A., Sun J., Bozym D.J., Zhang W., Luoma A., Giobbie-Hurder A., Peter L. (2017). An immunogenic personal neoantigen vaccine for patients with melanoma. Nature.

[89] Weber J.S., Carlino M.S., Khattak A., Meniawy T., Ansstas G., Taylor M.H., Kim K.B., McKean M., Long G.V., Sullivan R.J. (2024). Individualised neoantigen therapy mRNA-4157 (V940) plus pembrolizumab versus pembrolizumab monotherapy in resected melanoma (KEYNOTE-942): A randomised, phase 2b study. Lancet.

[90] Liu J., Fu M., Wang M., Wan D., Wei Y., Wei X. (2022). Cancer vaccines as promising immuno-therapeutics: Platforms and current progress. J. Hematol. Oncol..

[91] Wherry E.J., Kurachi M. (2015). Molecular and cellular insights into T cell exhaustion. Nat. Rev. Immunol..

[92] Chen W., Zhang Z., Zhang S., Zhu P., Ko J.K.-S., Yung K.K.-L. (2021). MUC1: Structure, Function, and Clinic Application in Epithelial Cancers. Int. J. Mol. Sci..

[93] Saltos A., Khalil F., Smith M., Li J., Schell M., Antonia S.J., Gray J.E. (2018). Clinical associations of mucin 1 in human lung cancer and precancerous lesions. Oncotarget.

[94] Beatty P.L., Narayanan S., Gariépy J., Ranganathan S., Finn O.J. (2010). Vaccine against MUC1 antigen expressed in inflammatory bowel disease and cancer lessens colonic inflammation and prevents progression to colitis-associated colon cancer. Cancer Prev. Res..

[95] Murwanti R., Denda-Nagai K., Sugiura D., Mogushi K., Gendler S.J., Irimura T. (2023). Prevention of Inflammation-Driven Colon Carcinogenesis in Human MUC1 Transgenic Mice by Vaccination with MUC1 DNA and Dendritic Cells. Cancers.

[96] Kimura T., McKolanis J.R., Dzubinski L.A., Islam K., Potter D.M., Salazar A.M., Schoen R.E., Finn O.J. (2013). MUC1 vaccine for individuals with advanced adenoma of the colon: A cancer immunoprevention feasibility study. Cancer Prev. Res..

[97] Lohmueller J.J., Sato S., Popova L., Chu I.M., Tucker M.A., Barberena R., Innocenti G.M., Cudic M., Ham J.D., Cheung W.C. (2016). Antibodies elicited by the first non-viral prophylactic cancer vaccine show tumor-specificity and immunotherapeutic potential. Sci. Rep..

[98] Schoen R.E., Boardman L.A., Cruz-Correa M., Bansal A., Kastenberg D., Hur C., Dzubinski L., Kaufman S.F., Rodriguez L.M., Richmond E. (2023). Randomized, Double-Blind, Placebo-Controlled Trial of MUC1 Peptide Vaccine for Prevention of Recurrent Colorectal Adenoma. Clin. Cancer Res..

[99] Chu X., Tian W., Ning J., Xiao G., Zhou Y., Wang Z., Zhai Z., Tanzhu G., Yang J., Zhou R. (2024). Cancer stem cells: Advances in knowledge and implications for cancer therapy. Signal Transduct. Target. Ther..

[100] Quaglino E., Conti L., Cavallo F. (2020). Breast cancer stem cell antigens as targets for immunotherapy. Semin. Immunol..

[101] Lanzardo S., Conti L., Rooke R., Ruiu R., Accart N., Bolli E., Arigoni M., Macagno M., Barrera G., Pizzimenti S. (2016). Immunotargeting of Antigen xCT Attenuates Stem-like Cell Behavior and Metastatic Progression in Breast Cancer. Cancer Res..

[102] Witt K., Ligtenberg M.A., Conti L., Lanzardo S., Ruiu R., Wallmann T., Tufvesson-Stiller H., Chambers B.J., Rolny C., Lladser A. (2018). Cripto-1 Plasmid DNA Vaccination Targets Metastasis and Cancer Stem Cells in Murine Mammary Carcinoma. Cancer Immunol. Res..

[103] Bolli E., O’Rourke J.P., Conti L., Lanzardo S., Rolih V., Christen J.M., Barutello G., Forni M., Pericle F., Cavallo F. (2018). A Virus-Like-Particle immunotherapy targeting Epitope-Specific anti-xCT expressed on cancer stem cell inhibits the progression of metastatic cancer in vivo. Oncoimmunology.

[104] Donofrio G., Tebaldi G., Lanzardo S., Ruiu R., Bolli E., Ballatore A., Rolih V., Macchi F., Conti L., Cavallo F. (2018). Bovine herpesvirus 4-based vector delivering the full length xCT DNA efficiently protects mice from mammary cancer metastases by targeting cancer stem cells. Oncoimmunology.

[105] Rolih V., Caldeira J., Bolli E., Salameh A., Conti L., Barutello G., Riccardo F., Magri J., Lamolinara A., Parra K. (2020). Development of a VLP-Based Vaccine Displaying an xCT Extracellular Domain for the Treatment of Metastatic Breast Cancer. Cancers.

[106] Finn O.J., Ward J., Krpata T., Fatis S., McKolanis J., Xue J., Beatty P., Jacqueline C., Kaufman S., Akerley C. (2023). Abstract PR002: A pilot study of a MUC1 vaccine in current and former smokers at high risk for lung cancer. Cancer Prev. Res..

[107] Domchek S.M., Torres A., Aaron M., Miller J., Seger P., Knollman H.M., Reiss K.A., Shah P.D., Morrow M.P., Skolnik J. (2025). Phase Ib study of a plasmid DNA–based immunotherapy encoding the hTERT, PSMA, and WT1 (INO-5401) +/− IL12 (INO-9012) followed by electroporation in cancer patients and healthy individuals with *BRCA1/2* mutations. J. Clin. Oncol..

[108] Haldar S.D., Huff A., Diwan E.A., Ferguson A., Judkins C., Lu J., Wang H., Sinan H., Thoburn C., Bever K.M. (2024). Abstract CT022: Mutant KRAS peptide-based vaccine in patients at high risk of developing pancreatic cancer: Preliminary analysis from a phase I study. Cancer Res..

[109] Haldar S.D., Judkins C., Ferguson A., Abou Diwan E., Lim S.J., Wang H., Nauroth J., Goggins M., Laheru D., Jaffee E.M. (2023). A phase I study of a mutant KRAS-targeted long peptide vaccine in patients at high risk of developing pancreatic cancer. J. Clin. Oncol..

[110] Vilar E., Willis J., D’Alise M., Hall M., Cruz-Correa M., Idos G.E., Thirumurthi S., Leoni G., Garzia I., Antonucci L. (2024). 638 Nous-209 vaccine induces shared neoantigen immunogenicity for cancer interception in healthy lynch syndrome carriers: Results from phase Ib/II trial. J. Immunother. Cancer.

[111] Willis J., D’Alise A.M., Hall M.J., Cruz-Correa M., Idos G.E., Thirumurthi S., Ballester V., Leoni G., Garzia I., Antonucci L. (2025). Abstract 6427: Nous-209 off-the-shelf neoantigen immunotherapy induces robust neoantigen T cell response with the potential to intercept cancer in Lynch syndrome carriers. Cancer Res..

[112] Peng M., Mo Y., Wang Y., Wu P., Zhang Y., Xiong F., Guo C., Wu X., Li Y., Li X. (2019). Neoantigen vaccine: An emerging tumor immunotherapy. Mol. Cancer.

[113] Hu Z., Guo X., Li Z., Meng Z., Huang S. (2024). The neoantigens derived from transposable elements—A hidden treasure for cancer immunotherapy. Biochim. Biophys. Acta BBA—Rev. Cancer.

[114] Turajlic S., Litchfield K., Xu H., Rosenthal R., McGranahan N., Reading J.L., Wong Y.N.S., Rowan A., Kanu N., Al Bakir M. (2017). Insertion-and-deletion-derived tumour-specific neoantigens and the immunogenic phenotype: A pan-cancer analysis. Lancet Oncol..

[115] Wei Z., Zhou C., Zhang Z., Guan M., Zhang C., Liu Z., Liu Q. (2019). The Landscape of Tumor Fusion Neoantigens: A Pan-Cancer Analysis. iScience.

[116] Van Dorst M.M.A.R., Pyuza J.J., Nkurunungi G., Kullaya V.I., Smits H.H., Hogendoorn P.C.W., Wammes L.J., Everts B., Elliott A.M., Jochems S.P. (2024). Immunological factors linked to geographical variation in vaccine responses. Nat. Rev. Immunol..

[117] Bolivar A.M., Duzagac F., Sinha K.M., Vilar E. (2023). Advances in vaccine development for cancer prevention and treatment in Lynch Syndrome. Mol. Aspects Med..

[118] Pastor D.M., Schlom J. (2021). Immunology of Lynch Syndrome. Curr. Oncol. Rep..

[119] Gebert J., Gelincik O., Oezcan-Wahlbrink M., Marshall J.D., Hernandez-Sanchez A., Urban K., Long M., Cortes E., Tosti E., Katzenmaier E.-M. (2021). Recurrent Frameshift Neoantigen Vaccine Elicits Protective Immunity with Reduced Tumor Burden and Improved Overall Survival in a Lynch Syndrome Mouse Model. Gastroenterology.

[120] Solomon A., Alteber Z., Bassan D., Sharbi-Yunger A., Esbit S., Tzehoval E., Eisenbach L. (2022). On the development of a neoantigen vaccine for the prevention of Lynch Syndrome. Int. J. Cancer.

[121] Kloor M., Reuschenbach M., Pauligk C., Karbach J., Rafiyan M.-R., Al-Batran S.-E., Tariverdian M., Jäger E., von Knebel Doeberitz M. (2020). A Frameshift Peptide Neoantigen-Based Vaccine for Mismatch Repair-Deficient Cancers: A Phase I/IIa Clinical Trial. Clin. Cancer Res..

[122] Fisher B., Dignam J., Wolmark N., Wickerham D.L., Fisher E.R., Mamounas E., Smith R., Begovic M., Dimitrov N.V., Margolese R.G. (1999). Tamoxifen in treatment of intraductal breast cancer: National Surgical Adjuvant Breast and Bowel Project B-24 randomised controlled trial. Lancet.

[123] Jankowski J.A.Z., De Caestecker J., Love S.B., Reilly G., Watson P., Sanders S., Ang Y., Morris D., Bhandari P., Brooks C. (2018). Esomeprazole and aspirin in Barrett’s oesophagus (AspECT): A randomised factorial trial. Lancet.

[124] The FUTURE II Study Group (2007). Quadrivalent Vaccine against Human Papillomavirus to Prevent High-Grade Cervical Lesions. N. Engl. J. Med..

[125] Sandler R.S., Halabi S., Baron J.A., Budinger S., Paskett E., Keresztes R., Petrelli N., Pipas J.M., Karp D.D., Loprinzi C.L. (2003). A Randomized Trial of Aspirin to Prevent Colorectal Adenomas in Patients with Previous Colorectal Cancer. N. Engl. J. Med..

[126] Reid B.J., Prevo L.J., Galipeau P.C., Sanchez C.A., Longton G., Levine D.S., Blount P.L., Rabinovitch P.S. (2001). Predictors of progression in Barrett’s esophagus II: Baseline 17p (p53) loss of heterozygosity identifies a patient subset at increased risk for neoplastic progression. Am. J. Gastroenterol..

[127] Dave K., Ali A., Magalhaes M. (2020). Increased expression of PD-1 and PD-L1 in oral lesions progressing to oral squamous cell carcinoma: A pilot study. Sci. Rep..

[128] Rangel R., Pickering C.R., Sikora A.G., Spiotto M.T. (2022). Genetic Changes Driving Immunosuppressive Microenvironments in Oral Premalignancy. Front. Immunol..

[129] Iyer P.G., Codipilly D.C., Chandar A.K., Agarwal S., Wang K.K., Leggett C.L., Latuche L.R., Schulte P.J. (2022). Prediction of Progression in Barrett’s Esophagus Using a Tissue Systems Pathology Test: A Pooled Analysis of International Multicenter Studies. Clin. Gastroenterol. Hepatol..

[130] Campos-Carrillo A., Weitzel J.N., Sahoo P., Rockne R., Mokhnatkin J.V., Murtaza M., Gray S.W., Goetz L., Goel A., Schork N. (2020). Circulating tumor DNA as an early cancer detection tool. Pharmacol. Ther..

[131] Pauwels E.K.J., Bourguignon M.H. (2022). PARP Inhibition and Beyond in BRCA-Associated Breast Cancer in Women: A State-Of-The-Art Summary of Preclinical Research on Risk Reduction and Clinical Benefits. Med. Princ. Pract. Int. J. Kuwait Univ. Health Sci. Cent..

[132] Botros M., de Boer O.J., Cardenas B., Bekkers E.J., Jansen M., van der Wel M.J., Sánchez C.I., Meijer S.L. (2024). Deep Learning for Histopathological Assessment of Esophageal Adenocarcinoma Precursor Lesions. Mod. Pathol..

[133] Maldonado F., Duan F., Raghunath S.M., Rajagopalan S., Karwoski R.A., Garg K., Greco E., Nath H., Robb R.A., Bartholmai B.J. (2015). Noninvasive Computed Tomography-based Risk Stratification of Lung Adenocarcinomas in the National Lung Screening Trial. Am. J. Respir. Crit. Care Med..

[134] Maldonado F., Boland J.M., Raghunath S., Aubry M.C., Bartholmai B.J., Deandrade M., Hartman T.E., Karwoski R.A., Rajagopalan S., Sykes A.-M. (2013). Noninvasive characterization of the histopathologic features of pulmonary nodules of the lung adenocarcinoma spectrum using computer-aided nodule assessment and risk yield (CANARY)—A pilot study. J. Thorac. Oncol..

[135] DeWard A., Critchley-Thorne R.J., Von Stechow L. (2018). Systems Biology Approaches in Cancer Pathology. Cancer Systems Biology.

[136] Prichard J.W., Davison J.M., Campbell B.B., Repa K.A., Reese L.M., Nguyen X.M., Li J., Foxwell T., Taylor D.L., Critchley-Thorne R.J. (2015). TissueCypher^TM^: A systems biology approach to anatomic pathology. J. Pathol. Inform..

[137] Critchley-Thorne R.J., Duits L.C., Prichard J.W., Davison J.M., Jobe B.A., Campbell B.B., Zhang Y., Repa K.A., Reese L.M., Li J. (2016). A Tissue Systems Pathology Assay for High-Risk Barrett’s Esophagus. Cancer Epidemiol. Biomarkers Prev..

[138] Critchley-Thorne R.J., Davison J.M., Prichard J.W., Reese L.M., Zhang Y., Repa K., Li J., Diehl D.L., Jhala N.C., Ginsberg G.G. (2017). A Tissue Systems Pathology Test Detects Abnormalities Associated with Prevalent High-Grade Dysplasia and Esophageal Cancer in Barrett’s Esophagus. Cancer Epidemiol. Biomarkers Prev..

[139] Davison J.M., Goldblum J., Grewal U.S., McGrath K., Fasanella K., Deitrick C., DeWard A.D., Bossart E.A., Hayward S.L., Zhang Y. (2020). Independent Blinded Validation of a Tissue Systems Pathology Test to Predict Progression in Patients with Barrett’s Esophagus. Am. J. Gastroenterol..

[140] Frei N.F., Khoshiwal A.M., Konte K., Bossart E.A., Stebbins K., Zhang Y., Pouw R.E., Ten Kate F.J.W., Seldenrijk K.A., Meijer S.L. (2021). Tissue Systems Pathology Test Objectively Risk Stratifies Barrett’s Esophagus Patients with Low-Grade Dysplasia. Am. J. Gastroenterol..

[141] Diehl D.L., Khara H.S., Akhtar N., Critchley-Thorne R.J. (2021). TissueCypher Barrett’s esophagus assay impacts clinical decisions in the management of patients with Barrett’s esophagus. Endosc. Int. Open.

[142] Srivastava S., Wagner P.D., Hughes S.K., Ghosh S. (2023). PreCancer Atlas: Present and Future. Cancer Prev. Res..

[143] De Bruijn I., Nikolov M., Lau C., Clayton A., Gibbs D.L., Mitraka E., Pozhidayeva D., Lash A., Sumer S.O., Altreuter J. (2025). Sharing data from the Human Tumor Atlas Network through standards, infrastructure and community engagement. Nat. Methods.

[144] Rozenblatt-Rosen O., Regev A., Oberdoerffer P., Nawy T., Hupalowska A., Rood J.E., Ashenberg O., Cerami E., Coffey R.J., Demir E. (2020). The Human Tumor Atlas Network: Charting Tumor Transitions across Space and Time at Single-Cell Resolution. Cell.

[145] Chen B., Scurrah C.R., McKinley E.T., Simmons A.J., Ramirez-Solano M.A., Zhu X., Markham N.O., Heiser C.N., Vega P.N., Rolong A. (2021). Differential pre-malignant programs and microenvironment chart distinct paths to malignancy in human colorectal polyps. Cell.

[146] Heiser C.N., Simmons A.J., Revetta F., McKinley E.T., Ramirez-Solano M.A., Wang J., Kaur H., Shao J., Ayers G.D., Wang Y. (2023). Molecular cartography uncovers evolutionary and microenvironmental dynamics in sporadic colorectal tumors. Cell.

[147] Islam M., Yang Y., Simmons A.J., Shah V.M., Musale K.P., Xu Y., Tasneem N., Chen Z., Trinh L.T., Molina P. (2024). Temporal recording of mammalian development and precancer. Nature.

[148] Becker W.R., Nevins S.A., Chen D.C., Chiu R., Horning A.M., Guha T.K., Laquindanum R., Mills M., Chaib H., Ladabaum U. (2022). Single-cell analyses define a continuum of cell state and composition changes in the malignant transformation of polyps to colorectal cancer. Nat. Genet..

[149] Esplin E.D., Hanson C., Wu S., Horning A.M., Barapour N., Nevins S.A., Jiang L., Contrepois K., Lee H., Guha T.K. (2024). Multiomic analysis of familial adenomatous polyposis reveals molecular pathways associated with early tumorigenesis. Nat. Cancer.

[150] Zhu Y., Lee H., White S., Weimer A.K., Monte E., Horning A., Nevins S.A., Esplin E.D., Paul K., Krieger G. (2024). Global loss of promoter–enhancer connectivity and rebalancing of gene expression during early colorectal cancer carcinogenesis. Nat. Cancer.

[151] Gindra R.H., Zheng Y., Green E.J., Reid M.E., Mazzilli S.A., Merrick D.T., Burks E.J., Kolachalama V.B., Beane J.E. (2024). Graph Perceiver Network for Lung Tumor and Bronchial Premalignant Lesion Stratification from Histopathology. Am. J. Pathol..

[152] Gómez-López S., Alhendi A.S.N., Przybilla M.J., Bordeu I., Whiteman Z.E., Butler T., Rouhani M.J., Kalinke L., Uddin I., Otter K.E.J. (2025). Aberrant basal cell clonal dynamics shape early lung carcinogenesis. Science.

[153] Yanagawa J., Tran L.M., Salehi-Rad R., Lim R.J., Dumitras C., Fung E., Wallace W.D., Prosper A.E., Fishbein G., Shea C. (2023). Single-Cell Characterization of Pulmonary Nodules Implicates Suppression of Immunosurveillance across Early Stages of Lung Adenocarcinoma. Cancer Res..

[154] Qin X., Strand S.H., Lee M.R., Saraswathibhatla A., van IJzendoorn D.G.P., Zhu C., Vennam S., Varma S., Hall A., Factor R.E. (2024). Single Cell Expression Analysis of Ductal Carcinoma in Situ Identifies Complex Genotypic-Phenotypic Relationships Altering Epithelial Composition. BioRxiv.

[155] Risom T., Glass D.R., Averbukh I., Liu C.C., Baranski A., Kagel A., McCaffrey E.F., Greenwald N.F., Rivero-Gutiérrez B., Strand S.H. (2022). Transition to invasive breast cancer is associated with progressive changes in the structure and composition of tumor stroma. Cell.

[156] Strand S.H., Rivero-Gutiérrez B., Houlahan K.E., Seoane J.A., King L.M., Risom T., Simpson L.A., Vennam S., Khan A., Cisneros L. (2022). Molecular classification and biomarkers of clinical outcome in breast ductal carcinoma in situ: Analysis of TBCRC 038 and RAHBT cohorts. Cancer Cell.

[157] Strand S.H., Houlahan K.E., Branch V., King L.M., Lynch T., Rivero-Guitiérrez B., Harmon B., Couch F., Gallagher K., Kilgore M. (2024). Analysis of ductal carcinoma in situ by self-reported race reveals molecular differences related to outcome. Breast Cancer Res. BCR.

[158] Nirmal A.J., Maliga Z., Vallius T., Quattrochi B., Chen A.A., Jacobson C.A., Pelletier R.J., Yapp C., Arias-Camison R., Chen Y.-A. (2022). The Spatial Landscape of Progression and Immunoediting in Primary Melanoma at Single-Cell Resolution. Cancer Discov..

[159] Yapp C., Nirmal A.J., Zhou F., Wong A.Y.H., Tefft J.B., Lu Y.D., Shang Z., Maliga Z., Montero Llopis P., Murphy G.F. (2023). Highly Multiplexed 3D Profiling of Cell States and Immune Niches in Human Tumours 2023. Nat. Methods.

[160] Cui Zhou D., Jayasinghe R.G., Chen S., Herndon J.M., Iglesia M.D., Navale P., Wendl M.C., Caravan W., Sato K., Storrs E. (2022). Spatially restricted drivers and transitional cell populations cooperate with the microenvironment in untreated and chemo-resistant pancreatic cancer. Nat. Genet..

[161] Srivastava S., Ghosh S., Kagan J., Mazurchuk R., Boja E., Chuaqui R., Chavarria-Johnson E., Davidsen T., Eary J., Haim T. (2018). The Making of a PreCancer Atlas: Promises, Challenges, and Opportunities. Trends Cancer.

[162] Serrano D., Gandini S., Thomas P., Crew K.D., Kumar N.B., Vornik L.A., Lee J.J., Veronesi P., Viale G., Guerrieri-Gonzaga A. (2023). Efficacy of Alternative Dose Regimens of Exemestane in Postmenopausal Women with Stage 0 to II Estrogen Receptor–Positive Breast Cancer: A Randomized Clinical Trial. JAMA Oncol..

[163] Desravines N., Miele K., Carlson R., Chibwesha C., Rahangdale L. (2020). Topical therapies for the treatment of cervical intraepithelial neoplasia (CIN) 2–3: A narrative review. Gynecol. Oncol. Rep..

[164] Maiman M. (1999). Vaginal 5-fluorouracil for high-grade cervical dysplasia in human immunodeficiency virus infection: A randomized trial. Obstet. Gynecol..

[165] Rahangdale L., Lippmann Q.K., Garcia K., Budwit D., Smith J.S., Van Le L. (2014). Topical 5-fluorouracil for treatment of cervical intraepithelial neoplasia 2: A randomized controlled trial. Am. J. Obstet. Gynecol..

[166] Al Rabadi L., Bergan R. (2017). A Way Forward for Cancer Chemoprevention: Think Local. Cancer Prev. Res..

[167] Jansen M.H.E., Kessels J.P.H.M., Nelemans P.J., Kouloubis N., Arits A.H.M.M., Van Pelt H.P.A., Quaedvlieg P.J.F., Essers B.A.B., Steijlen P.M., Kelleners-Smeets N.W.J. (2019). Randomized Trial of Four Treatment Approaches for Actinic Keratosis. N. Engl. J. Med..

[168] Rosenberg A.R., Tabacchi M., Ngo K.H., Wallendorf M., Rosman I.S., Cornelius L.A., Demehri S. (2019). Skin cancer precursor immunotherapy for squamous cell carcinoma prevention. JCI Insight.

[169] Azin M., Oka T., Hsu C.P., Malo J., Safa K., Curiel-Lewandrowski C.N., Anadkat M.J., Kulkarni R.P., House M., Bauman J.E. (2025). 0426 UAZ22-10-01: A Phase IIa single-arm open-label clinical trial of calcipotriene plus 5-fluorouracil immunotherapy for skin cancer prevention in organ transplant recipients. J. Investig. Dermatol..

[170] Bakhrushina E.O., Shumkova M.M., Avdonina Y.V., Ananian A.A., Babazadeh M., Pouya G., Grikh V.V., Zubareva I.M., Kosenkova S.I., Krasnyuk I.I. (2025). Transdermal Drug Delivery Systems: Methods for Enhancing Skin Permeability and Their Evaluation. Pharmaceutics.

[171] Lee O., Page K., Ivancic D., Helenowski I., Parini V., Sullivan M.E., Margenthaler J.A., Chatterton R.T., Jovanovic B., Dunn B.K. (2014). A randomized phase II presurgical trial of transdermal 4-hydroxytamoxifen gel versus oral tamoxifen in women with ductal carcinoma in situ of the breast. Clin. Cancer Res..

[172] Khan S.A., Mi X., Xu Y., Blanco L.Z., Akasha A.M., Pilewskie M., Degnim A.C., AlHilli Z., Amin A.L., Hwang E.S. (2023). Presurgical Oral Tamoxifen vs Transdermal 4-Hydroxytamoxifen in Women with Ductal Carcinoma In Situ: A Randomized Clinical Trial. JAMA Surg..

[173] Fonseca B.O., Possati-Resende J.C., Salcedo M.P., Schmeler K.M., Accorsi G.S., Fregnani J.H.T.G., Antoniazzi M., Pantano N.P., Santana I.V.V., Matsushita G.M. (2021). Topical Imiquimod for the Treatment of High-Grade Squamous Intraepithelial Lesions of the Cervix: A Randomized Controlled Trial. Obstet. Gynecol..

[174] Snoeck R., Noel J.-C., Muller C., De Clercq E., Bossens M. (2000). Cidofovir, a new approach for the treatment of cervix intraepithelial neoplasia grade III (CIN III). J. Med. Virol..

[175] Buck H.W., Guth K.J. (2003). Treatment of vaginal intraepithelial neoplasia (primarily low grade) with imiquimod 5% cream. J. Low. Genit. Tract Dis..

[176] Sasagasako N., Kosaka K., Sagae Y., Itoh K., Aratake J., Yamada K., Inayama Y., Gou R., Kawamura A., Yamanishi M. (2020). Recurrent vaginal intraepithelial neoplasia successfully treated with topical imiquimod: A case report. Mol. Clin. Oncol..

[177] Simões A.C., Sarmento A.C., Aquino A.C., Eleutério J., Do Val Guimarães I.C., Falsetta M.L., Gonçalves A.K. (2025). Treatment Interventions for Usual-Type Vulvar Intraepithelial Neoplasia: A Systematic Review and Meta-analysis. J. Low. Genit. Tract Dis..

[178] Tristram A., Hurt C.N., Madden T., Powell N., Man S., Hibbitts S., Dutton P., Jones S., Nordin A.J., Naik R. (2014). Activity, safety, and feasibility of cidofovir and imiquimod for treatment of vulval intraepithelial neoplasia (RT3VIN): A multicentre, open-label, randomised, phase 2 trial. Lancet Oncol..

[179] Weis S. (2013). Current treatment options for management of anal intraepithelial neoplasia. OncoTargets Ther..

[180] Chau L., Jabara J.T., Lai W., Svider P.F., Warner B.M., Lin H.-S., Raza S.N., Fribley A.M. (2017). Topical agents for oral cancer chemoprevention: A systematic review of the literature. Oral Oncol..

[181] Gorsky M., Epstein J.B. (2002). The effect of retinoids on premalignant oral lesions: Focus on topical therapy. Cancer.

[182] Terlou A., van Seters M., Ewing P.C., Aaronson N.K., Gundy C.M., Heijmans-Antonissen C., Quint W.G.V., Blok L.J., van Beurden M., Helmerhorst T.J.M. (2011). Treatment of vulvar intraepithelial neoplasia with topical imiquimod: Seven years median follow-up of a randomized clinical trial. Gynecol. Oncol..

[183] Hendriks N., Koeneman M.M., van de Sande A.J.M., Penders C.G.J., Piek J.M.J., Kooreman L.F.S., van Kuijk S.M.J., Hoosemans L., Sep S.J.S., de Vos Van Steenwijk P.J. (2022). Topical Imiquimod Treatment of High-grade Cervical Intraepithelial Neoplasia (TOPIC-3): A Nonrandomized Multicenter Study. J. Immunother..

[184] Deljavan Ghodrati A., Çomoğlu T. (2024). MUCOADHESIVE POLYMERS IN COLON TARGETED DRUG DELIVERY SYSTEMS: A COMPREHENSIVE REVIEW. Ank. Univ. Eczacilik Fak. Derg..

[185] Duggan S., Cummins W., O’ Donovan O., Hughes H., Owens E. (2017). Thiolated polymers as mucoadhesive drug delivery systems. Eur. J. Pharm. Sci..

[186] Shantha K.L., Ravichandran P., Rao K.P. (1995). Azo polymeric hydrogels for colon targeted drug delivery. Biomaterials.

[187] Abbasi M., Sohail M., Minhas M.U., Mahmood A., Shah S.A., Munir A., Kashif M.-U.-R. (2023). Folic acid-decorated alginate nanoparticles loaded hydrogel for the oral delivery of diferourylmethane in colorectal cancer. Int. J. Biol. Macromol..

[188] Philip A.K., Philip B. (2010). Colon targeted drug delivery systems: A review on primary and novel approaches. Oman Med. J..

[189] Piotrowska U., Orzechowska K. (2024). Advances in Chitosan-Based Smart Hydrogels for Colorectal Cancer Treatment. Pharmaceuticals.

[190] Noreen S., Pervaiz F., Ijaz M., Hanif M.F., Shoukat H., Maqbool I., Ashraf M.A., Mahmood H. (2025). Novel thiol-functionalized hyaluronic acid-based pH-responsive hydrogel: A promising mucoadhesive drug delivery approach toward the treatment of colorectal cancer. Colloids Surf. B Biointerfaces.

[191] Fernández-García R., Fraguas-Sánchez A.I. (2024). Nanomedicines for Pulmonary Drug Delivery: Overcoming Barriers in the Treatment of Respiratory Infections and Lung Cancer. Pharmaceutics.

[192] Brooks A.D., Tong W., Benedetti F., Kaneda Y., Miller V., Warrell R.P. (2000). Inhaled aerosolization of all-trans-retinoic acid for targeted pulmonary delivery. Cancer Chemother. Pharmacol..

[193] Dahl A.R., Grossi I.M., Houchens D.P., Scovell L.J., Placke M.E., Imondi A.R., Stoner G.D., De Luca L.M., Wang D., Mulshine J.L. (2000). Inhaled isotretinoin (13-cis retinoic acid) is an effective lung cancer chemopreventive agent in A/J mice at low doses: A pilot study. Clin. Cancer Res..

[194] Wattenberg L.W., Wiedmann T.S., Estensen R.D., Zimmerman C.L., Steele V.E., Kelloff G.J. (1997). Chemoprevention of pulmonary carcinogenesis by aerosolized budesonide in female A/J mice. Cancer Res..

[195] Yan Y., Cook J., McQuillan J., Zhang G., Hitzman C.J., Wang Y., Wiedmann T.S., You M. (2007). Chemopreventive effect of aerosolized polyphenon E on lung tumorigenesis in A/J mice. Neoplasia.

[196] Lam S., leRiche J.C., McWilliams A., Macaulay C., Dyachkova Y., Szabo E., Mayo J., Schellenberg R., Coldman A., Hawk E. (2004). A randomized phase IIb trial of pulmicort turbuhaler (budesonide) in people with dysplasia of the bronchial epithelium. Clin. Cancer Res..

[197] Veronesi G., Szabo E., Decensi A., Guerrieri-Gonzaga A., Bellomi M., Radice D., Ferretti S., Pelosi G., Lazzeroni M., Serrano D. (2011). Randomized phase II trial of inhaled budesonide versus placebo in high-risk individuals with CT screen-detected lung nodules. Cancer Prev. Res..

[198] Good L.M., Miller M.D., High W.A. (2011). Intralesional agents in the management of cutaneous malignancy: A review. J. Am. Acad. Dermatol..

[199] Gulley J.L., Heery C.R., Madan R.A., Walter B.A., Merino M.J., Dahut W.L., Tsang K.-Y., Schlom J., Pinto P.A. (2013). Phase I study of intraprostatic vaccine administration in men with locally recurrent or progressive prostate cancer. Cancer Immunol. Immunother. CII.

[200] Kim S., Woo Y.R., Cho S.H., Lee J.D., Kim H.S. (2024). Clinical Efficacy of 5-Fluorouracil and Bleomycin in Dermatology. J. Clin. Med..

[201] Love S.M., Zhang W., Gordon E.J., Rao J., Yang H., Li J., Zhang B., Wang X., Chen G., Zhang B. (2013). A feasibility study of the intraductal administration of chemotherapy. Cancer Prev. Res..

[202] Lu J.-L., Xia Q.-D., Lu Y.-H., Liu Z., Zhou P., Hu H.-L., Wang S.-G. (2020). Efficacy of intravesical therapies on the prevention of recurrence and progression of non-muscle-invasive bladder cancer: A systematic review and network meta-analysis. Cancer Med..

[203] Nel A., Van Niekerk N., Kapiga S., Bekker L.-G., Gama C., Gill K., Kamali A., Kotze P., Louw C., Mabude Z. (2016). Safety and Efficacy of a Dapivirine Vaginal Ring for HIV Prevention in Women. N. Engl. J. Med..

[204] Porten S.P., Leapman M.S., Greene K.L. (2015). Intravesical chemotherapy in non-muscle-invasive bladder cancer. Indian J. Urol. IJU.

[205] Saha I., Halder J., Rajwar T.K., Mahanty R., Pradhan D., Dash P., Das C., Rai V.K., Kar B., Ghosh G. (2024). Novel Drug Delivery Approaches for the Localized Treatment of Cervical Cancer. AAPS PharmSciTech.

[206] Choradia N., Szabo E. (2024). Repurposing Drugs for Cancer Prevention: Targeting Mechanisms Common to Chronic Diseases. Cancer J..

